# Metabolite Profiling of Triterpene Glycosides of the Far Eastern Sea Cucumber *Eupentacta fraudatrix* and Their Distribution in Various Body Components Using LC-ESI QTOF-MS

**DOI:** 10.3390/md15100302

**Published:** 2017-10-02

**Authors:** Roman S. Popov, Natalia V. Ivanchina, Alexandra S. Silchenko, Sergey A. Avilov, Vladimir I. Kalinin, Igor Yu. Dolmatov, Valentin A. Stonik, Pavel S. Dmitrenok

**Affiliations:** 1 G.B. Elyakov Pacific Institute of Bioorganic Chemistry, Far Eastern Branch of Russian Academy of Sciences, 159 Prospect 100-letiya Vladivostoka, Vladivostok 690022, Russia; prs_90@mail.ru (R.S.P.); ivanchina@piboc.dvo.ru (N.V.I.); sialexandra@mail.ru (A.S.S.); avilov-1957@mail.ru (S.A.A.); kalininv@piboc.dvo.ru (V.I.K.); stonik@piboc.dvo.ru (V.A.S.); 2 A.V. Zhirmunsky Institute of Marine Biology, National Scientific Center of Marine Biology, Far Eastern Branch of the Russian Academy of Sciences, 17 Palchevskogo St., Vladivostok 690041, Russia; idolmatov@mail.ru; 3 School of Natural Science, Far Eastern Federal University, 8 Sukhanova St., Vladivostok 690090, Russia

**Keywords:** sea cucumber, *Eupentacta fraudatrix*, triterpene glycoside, liquid chromatography–tandem mass spectrometry, metabolite profiling

## Abstract

The Far Eastern sea cucumber *Eupentacta fraudatrix* is an inhabitant of shallow waters of the south part of the Sea of Japan. This animal is an interesting and rich source of triterpene glycosides with unique chemical structures and various biological activities. The objective of this study was to investigate composition and distribution in various body components of triterpene glycosides of the sea cucumber *E. fraudatrix*. We applied LC-ESI MS (liquid chromatography–electrospray mass spectrometry) of whole body extract and extracts of various body components for metabolic profiling and structure elucidation of triterpene glycosides from the *E. fraudatrix*. Totally, 54 compounds, including 26 sulfated, 18 non-sulfated and 10 disulfated glycosides were detected and described. Triterpene glycosides from the body walls, gonads, aquapharyngeal bulbs, guts and respiratory trees were extracted separately and the distributions of the detected compounds in various body components were analyzed. Series of new glycosides with unusual structural features were described in *E. fraudatrix*, which allow clarifying the biosynthesis of these compounds. Comparison of the triterpene glycosides contents from the five different body components revealed that the profiles of triterpene glycosides were qualitatively similar, and only some quantitative variabilities for minor compounds were observed.

## 1. Introduction

Sea cucumbers (Class Holothuroidea, Phylum Echinodermata) are widespread slow-moving marine animals. Metabolome of these animals is characterized by the high content of triterpene glycosides of a great structural diversity. Triterpene glycosides of sea cucumbers have unique chemical structures, significantly differing from those of terrestrial plants. These compounds possess a variety of biological and pharmacological effects including cytotoxic [1,2], antifungal [3,4], bactericidal, hemolytic, antiviral and antiparasitic properties [5]. Some glycosides are capable to induce apoptosis, inhibit the growth of tumor cells [6] and have immunomodulatory properties [7]. In addition, certain species of sea cucumbers are a valuable maricultural resource [8].

Majority of triterpene glycosides from sea cucumbers possess a lanostane-type aglycone with an 18(20)-lactone called as holostane derivatives. Individual triterpene aglycones differ from each other in the number and arrangement of oxygen substituents, double bonds and in the structure of the side chains demonstrating a significant natural diversity. Some triterpene glycosides have aglycones with 18(16)-lactone or without a lactone cycle. Usually, triterpene glycosides have a polycyclic nucleus with 7(8)- or 9(11)-double bond and oxygen-containing substituents, which may be bonded to C-12, C-17 or C-16. The side chains of aglycones may have one or more double bonds, hydroxyl or acetate groups and other substituents. Some glycosides have aglycones with shortened side chains.

The oligosaccharide chain of triterpene glycosides is attached to C-3 of the aglycone and may include up to six sugar units. Oligosaccharide chains with up to four monosaccharide units usually have a linear structure, while the penta- and hexaosides contain a branching at the first or second monosaccharide unit. Xylose (Xyl), glucose (Glc), quinovose (Qui), 3-*O*-methylglucose (MeGlc), and, rarely, 3-*O*-methylxylose (MeXyl) are the most common sugar residues in triterpene glycosides. The first unit in oligosaccharide chain is always xylose, quinovose (rarely glucose or xylose) is usually the second monosaccharide unit and glucose (or xylose) is the third [1,4]. The methylated monosaccharides always occupy the terminal position. Many glycosides have up to three sulfate groups at certain positions of the oligosaccharide chain.

It is supposed that triterpene glycosides have multiple defensive roles such as defense against predators, parasites and microorganisms. Indeed, a high percentage of these compounds in the Cuvierian tubules, that can be ejected toward a predator as well as their strong ichthyotoxic and membranolytic effects indicate their effective action against predators [9,10]. In addition, it is likely that glycosides play an important role in regulating the reproduction of sea cucumbers [11].

Triterpene glycosides display taxonomic specificity for different systematic groups of sea cucumbers. The level of this specificity may be different in various sea cucumbers taxa. The glycoside structures are specific for one genus or a group of genera for the sea cucumbers of the order Aspidochirotida. However, these compounds are usually species-specific for the representatives of the order Dendrochirotida [12].

Triterpene glycosides are usually present in extracts of sea cucumbers as complex mixtures. Minor compounds of these extracts remain largely unstudied, although knowledge about their chemical structures is also important for understanding of biosynthesis and biological roles of these compounds. Recent investigations have demonstrated the power of MS approaches for profiling and for studying of the body distribution of sea cucumber triterpene glycosides [13,14]. All sea cucumbers contain triterpene glycosides in their body walls and viscera; however, recent studies have demonstrated difference in content of these metabolites in the Cuvierian tubules and in the body wall. All the glycosides, detected in the body walls of *Holothuria forskali* were found to be also present in the Cuvierian tubules but the latter also contain specific congeners. Furthermore, profiles of triterpene glycosides in both analyzed body parts varied under stress condition [9,10,15].

The sea cucumber *Eupentacta fraudatrix* (Djakonov et Baranova) (=*Cucumaria fraudatrix* Djakonov et Baranova = *Cucumaria obunca* Lampert) (Family Sclerodactylidae, Order Dendrochirotida) is a common species in the shallow waters of the south part of the Sea of Japan and is a rich source of triterpene glycosides. *E. fraudatrix* is a well-studied species that is often used as a model organism in biological studies of various processes such as development [16,17], regeneration [18,19,20] and immunity [21,22,23]. The previous investigations of the sea cucumber *E. fraudatrix* have led to isolation of 37 triterpene glycosides comprising 15 non-sulfated tetraosides (cucumariosides A_1_–A_15_ [24,25,26]), two non-sulfated triosides (cucumariosides B_1_ and B_2_ [27]), two non-sulfated pentaosides (cucumariosides C_1_ and C_2_ [28]), 4 sulfated tetraosides (cucumariosides G_1_–G_4_ [29,30,31,32]), eight sulfated pentaosides (cucumariosides H, H_2_–H_8_ [33,34,35]), two disulfated tetraosides (cucumariosides F_1_ and F_2_ [36]), and four disulfated pentaosides (cucumariosides I_1_–I_4_ [37,38]). The majority of the glycosides from *E. fraudatrix* are characterized by the presence of 3-*O*-MeXyl residue as terminal unit in carbohydrate chain, which is considered a chemotaxonomic marker of the genus *Eupentacta*. Herein, we describe the application of LC-ESI MS for metabolic profiling, evaluation of the structural variability, structure elucidation and further refinement of known biosynthetic patterns of triterpene glycosides from the sea cucumber *E. fraudatrix*. In addition, triterpene glycosides from the body walls, gonads, aquapharyngeal bulbs, guts and respiratory trees were extracted separately and distribution of the detected compounds in various body components were analyzed.

## 2. Results and Discussion

### 2.1. Profiling and Structural Identification of the Triterpene Glycosides from *E. fraudatrix*

**Figure 1:**
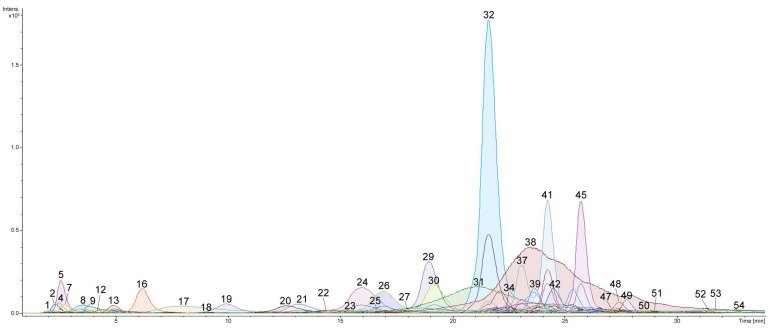
LC-ESI MS (liquid chromatography–electrospray mass spectrometry) total compounds chromatogram of detected triterpene glycosides in negative ion mode (sulfated, disulfated and non-sulfated glycosides were detected as [M − Na]^−^, [M − 2Na]^2−^ and [M − H]^−^ ions) in ethanol extract of sea cucumber *Eupentacta fraudatrix*.

**Figure 2:**
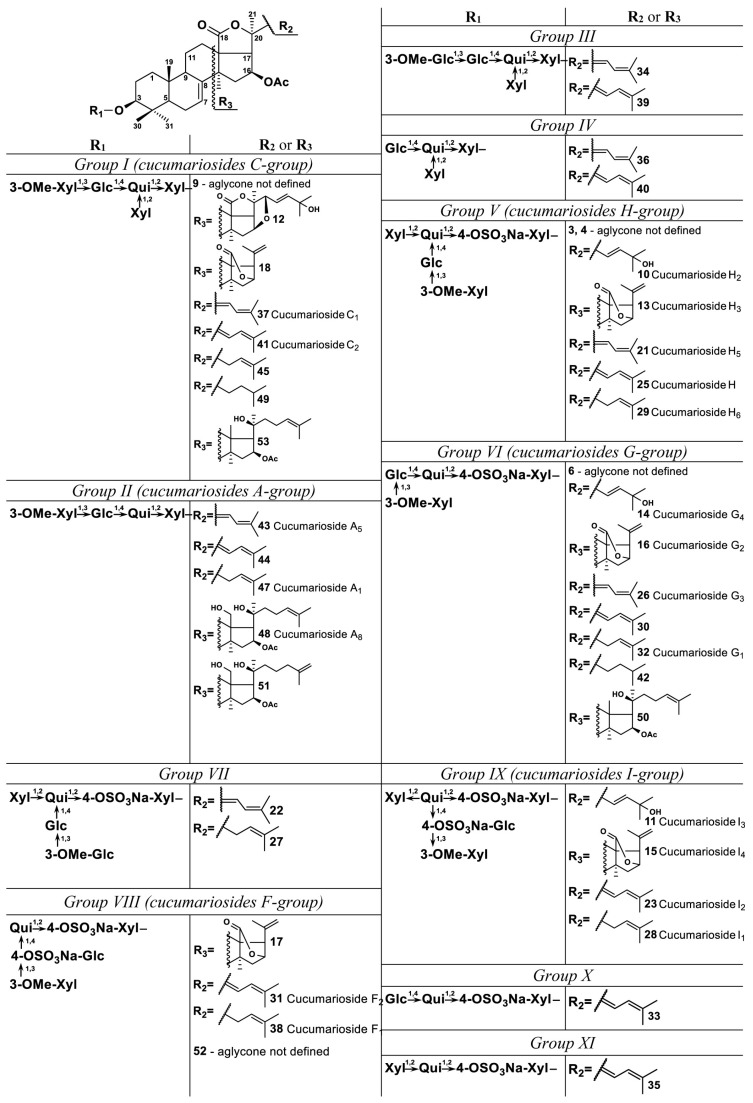
Structures of triterpene glycosides identified (**10**, **11**, **13**, **14**, **15**, **16**, **21**, **23**, **25**, **26**, **28**, **29**, **31**, **32**, **37**, **38**, **41**, **43**, **47**, and **48**) and proposed (**12**, **17**, **18**, **22**, **27**, **30**, **33**, **34**, **35**, **36**, **39**, **40**, **42**, **44**, **45**, **49**, **50**, **51**, and **53**) from the sea cucumber *E. fraudatrix* by LC–MS/MS method.

Profiling of triterpene glycosides from the whole body extract of the sea cucumber *E. fraudatrix* using LC-MS approach allowed numerous new as well as previously isolated triterpene glycosides to be characterized. The HPLC profile revealed at least 54 compounds, including 26 sulfated, 18 non-sulfated and 10 disulfated glycosides (Figure 1 and Figure 2; Table 1; Figure S1; the numbers of the compounds correspond to the peak numbers on (−)LC-MS chromatogram).

In positive ion mode, the majority of triterpene glycosides were detected within *m*/*z* range from 1000 to 1400 a.m.u. as [M + Na]^+^ ions. However, stable peaks of [M + Na]^+^ ions for disulfated glycosides were not observed in these conditions. In negative ion mode, sulfated and disulfated glycosides were detected as [M − Na]^−^ and [M − 2Na]^2−^ peaks, respectively, whereas non-sulfated compounds were detected as [M − H]^−^ peaks.

The assignments of these compounds in all samples analyzed were based on the data of high resolution LC-MS and LC-MS/MS performed in both negative and positive ion modes. Elemental composition, determined on the base of high resolution data (mass accuracy tolerance < 2 ppm), fragmentation patterns of MS and MS/MS spectra, as well as chromatographic behavior of the corresponding compounds, allowed their structures to be proposed. It is known that triterpene glycosides are characterized by large structural variability. Generally, mass spectrometry does not permit to determine configuration of the unknown compounds. Besides, different epimeric monosaccharides as well as types of bonds between sugars cannot be strictly distinguished only by MS. However, the combination of the obtained data and biosynthetic considerations allows tentative structural assignments for the detected compounds.

It is known that the majority of triterpene glycosides have a xylose at C-3 of the aglycone as the first monosaccharide unit, quinovose as the second monosaccharide unit, and glucose (or xylose) as the third monosaccharide unit in the main chain [1,4]. Methylated monosaccharides are always terminal units. Considering that the oligosaccharide chains of earlier isolated triterpene glycosides from this sea cucumber are closely related to each other and have general architecture 3-*O*-methyl-β-d-xylopyranosyl-(1→3)-β-d-glucopyranosyl-(1→4)-β-d-quinovopyranosyl-(1→2)-β-d-xylopyranosyl in the linear part of carbohydrate chains and may have β-d-xylopyranosyl-(1→2) in the branching at the second monosaccharide, some suggestions concerning structures of oligosaccharide chains of unknown glycosides may be proposed. In addition, most of the previously isolated glycosides of *E. fraudatrix* have a 16β-acetoxyholosta-7-ene aglycone. These common structural features give a possibility to propose structures for a series of newly identified glycosides.

**Figure 3:**
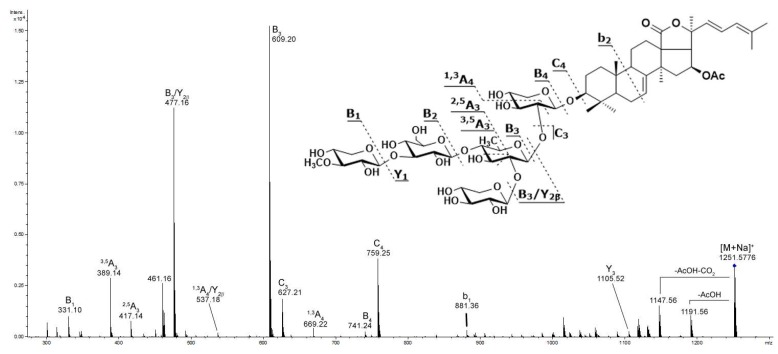
ESI MS/MS spectrum of [M + Na]^+^ precursor ion at *m*/*z* 1251 identified as cucumarioside C_2_ (**41**).

The structures of detected glycosides were characterized by tandem MS. The (−)MS/MS provided many product ion series arising from the cleavages of both glycosidic bonds and bond of aglycone side chain. (+)MS/MS provided an intense B- and C-type product ion series (nomenclature according to Domon and Costello [39]) arising from the cleavages of glycosidic bonds with charge located on saccharide fragment (Table S1). These product ion series are characteristic and provided information about the sequence of monosaccharide units in carbohydrate chains. For example, the positive product ion spectrum of [M + Na]^+^ precursor at *m*/*z* 1251.5776 (compound **41**) exhibits extensive fragmentation namely peaks at *m*/*z* 169.05 [MeXyl + Na]^+^, 331.10 [MeXyl + Glc + Na]^+^, 349.11 [MeXyl + Glc + H_2_O + Na]^+^, 389.14 [MeXyl + Glc + C_3_H_6_O + Na]^+^, 417.14 [MeXyl + Glc + C_4_H_6_O_2_ + Na]^+^, 477.16 [MeXyl + Glc + Qui + Na]^+^, 537.18 [MeXyl + Glc + Qui + C_2_H_4_O_2_ + Na]^+^, 609.20 [MeXyl + Glc + Qui + Xyl + Na]^+^, 627.21 [MeXyl + Glc + Qui + Xyl + H_2_O + Na]^+^, 669.22 [MeXyl + Glc + Qui + Xyl + C_2_H_4_O_2_ + Na]^+^, 741.24 [MeXyl + Glc + Qui + 2Xyl + Na]^+^, and 759.25 [MeXyl + Glc + Qui + 2Xyl + H_2_O + Na]^+^ (Figure 3). This fragmentation pattern corresponded to a branched non-sulfated oligosaccharide chain consisting of five monosaccharide units and compound **41** was identified as cucumarioside C_2_.

In some cases, several typical mass losses between the precursor and the fragment ions were detected in product ion spectra. These typical mass losses are related to the aglycone and provide information about the structure of nucleus and side chain. In MS/MS spectra of the majority of the glycosides, a mass loss of 60 Da between the precursor and the intense fragment ion was detected (for example, Figure S2). This corresponds to the loss of C_2_H_4_O_2_ molecule (acetic acid) and is a characteristic of the glycosides containing an acetoxy group [40]. Next intense fragment ion with a mass loss 104 Da from the precursor corresponds to the loss of a [C_2_H_4_O_2_ + CO_2_] fragment and is characteristic for the glycosides containing an acetoxy group and a 18(20)-lactone cycle. Analysis of the MS/MS spectra of a number of triterpene glycosides isolated earlier allowed to identify characteristic fragment peaks related with the cleavages of side chains. For example, (+)MS/MS spectra of the cucumarioside A_1_ with 24-ene side chain have a mass loss of 228.1359 Da, corresponding to the loss of a C_12_H_20_O_4_ fragment (Figure S2). The (+)MS/MS spectra of the glycosides with 22,24-diene system in the side chain display a characteristic mass loss of C_11_H_17_O_4_ fragments (213.1121 Da). Otherwise, spectra of cucumarioside A_15_ with saturated side chain display a characteristic mass losses of fragments C_12_H_22_O_4_ (230.1525 Da) and C_10_H_10_O_4_ (204.1370 Da). These fragmentation patterns enable to propose structural features of aglycones for newly identified glycosides.

In accordance with the structures of oligosaccharide chains, all detected glycosides of *E. fraudatrix* can be divided into eleven groups (I–XI). Compounds of group I (**9**, **12**, **18**, **37**, **41**, **45**, **49**, and **53**) had branched non-sulfated oligosaccharide chain consisting of five monosaccharide units—methylated xylose, glucose, quinovose and xylose in the main chain and xylose as branching unit at the second monosaccharide. Such type of oligosaccharide chain corresponds to known cucumariosides of the C-group. Obtained data allowed to identify the glycosides **37** (*m*/*z* 1227.5799 [M − H]^−^, calcd 1227.5804) as cucumarioside C_1_ and **41** (*m*/*z* 1227.5796 [M − H]^−^, calcd 1227.5804) as cucumarioside C_2_ [28]. These compounds have similar holostane type aglycones having 16β-OAc and 7(8)-double bond in the nucleus and two double bonds in side chains which differ in the configuration of C-22 (22*Z*,24-diene system for cucumarioside C_1_ and 22*E*,24-diene system for cucumarioside C_2_). As a result, these compounds have similar MS/MS spectra, but their retention times differ (Rt of **37** is 23.1 min, and Rt of **41**—24.2 min). It was found that the glycosides with 22*Z*,24-diene system in the side chain have shorter retention time then analogous compounds with 22*E*,24-diene system. This made it possible to identify such pairs of similar compounds. In addition, (+)MS/MS spectra of **37** and **41** have characteristic mass losses of 204 Da and 213 Da, which confirm their structure. Compounds **45** (*m*/*z* 1229.5956 [M − H]^−^, calcd 1229.5961) and **49** (*m*/*z* 1231.6110 [M − H]^−^, calcd 1231.6117) differed in 2 and 4 Da, respectively, versus compounds **37** and **41**. Their MS/MS spectra were similar to the spectra of compounds **37** and **41**, but in (+)MS/MS spectrum of **45** a characteristic fragment peak [M + Na − C_12_H_20_O_4_]^+^ was detected at *m*/*z* 1025.4557, and in (+)MS/MS spectrum of **49** the fragment peaks [M + Na − C_12_H_22_O_4_]^+^ and [M + Na − C_10_H_20_O_4_]^+^ were detected at *m*/*z* 1025.4550 and 1051.4709, respectively. This indicates the presence of one double bond in **45** and no double bonds in the side chain of **49**. All these data revealed that **45** has a 16β-acetoxyholosta-7,24-diene aglycone and **49** has 16β-acetoxyholosta-7-ene aglycone. Structures of these two compounds are similar to known cucumariosides A_1_ and A_15_, respectively, with additional xylose as branching monosaccharide unit. Aglycone of glycoside **18** (*m*/*z* 1101.5126 [M − H]^−^, calcd 1101.5123) has no fragmentation in tandem MS; HR MS and chromatographic behavior of this compound corresponds to that having a 23,24,25,26,27-pentanorlanostane aglycone with an 18(16)-lactone. Compound **12** (*m*/*z* 1201.5634 [M − H]^−^, calcd 1201.5648) may has 16*S*,22*R*-epoxy-holosta-7,23*E*-diene-25-ol aglycone. Such aglycone was previously found in cucumarioside H_8_, a sulfated derivative of **12** [33]. MS/MS data of **53** (*m*/*z* 1217.6314 [M − H]^−^, calcd 1217.6324) showed that this compound has an aglycone with one oxygen less than some other aglycones of this group. The presence of a characteristic fragment peak [M + Na − C_9_H_18_O]^+^ at *m*/*z* 1099.4918 may indicate that the aglycone of **53** has no γ-lactone moiety and probably has a structure of 16-acetoxy-20-hydroxy-lanosta-7,24-diene. The structure of the aglycone of glycoside **9** was not defined.

The compounds of group II (**43**, **44**, **47**, **48**, and **51**) have a linear non-sulfated oligosaccharide chain with four monosaccharides—methylated xylose, glucose, quinovose and xylose. Such type of oligosaccharide chain corresponds to known cucumariosides of the A-group. Glycoside **47** (*m*/*z* 1097.5534 [M − H]^−^, calcd 1097.5538) was identified as cucumarioside A_1_ based on comparison of its retention time, MS spectra and elemental composition with those of standard compound [24]. Glycoside **48** (*m*/*z* 1101.5851 [M − H]^−^, calcd 1101.5851) was identified as cucumarioside A_8_ by analogous way as for **47** [26]. The MS data obtained for compound **51** (*m*/*z* 1101.5843 [M − H]^−^, calcd 1101.5851) were identical of those of cucumarioside A_8_ indicating similarity of structures of **51** and **48**. Glycoside **51** may differ in configuration or position of the double bond in the side chain. We suggest that this glycoside contains a 20-hydroxy-25(26)-ene fragment in the aglycone. Glycoside **43** (*m*/*z* 1095.5380 [M − H]^−^, calcd 1095.5381) was identified as cucumarioside A_5_ based on MS spectra and elemental composition [24]. This metabolite has an aglycone with 22*Z*,24-diene system in the side chain. Compound **44** (*m*/*z* 1095.5379 [M − H]^−^, calcd 1095.5381) is similar to **43**, but its retention time is higher. This may indicate that **44** has an aglycone with 22*E*,24-diene system in the side chain.

Fragmentation of oligosaccharide chain of glycosides of the group III (**34** and **39**) was similar to fragmentation of oligosaccharide chain of glycosides of the group I, but all fragment peaks in MS/MS spectra were shifted by 30 Da (Table S1). This may be due to the replacement of the terminal methylated xylose with a methylated glucose residue. Thus, group III includes compounds having non-sulfated main oligosaccharide chain with methylated glucose, glucose, quinovose and xylose monosaccharide units and xylose as branching unit. Data of glycosides **34** (*m*/*z* 1257.5905 [M − H]^−^, calcd 1257.5910) and **39** (*m*/*z* 1257.5907 [M − H]^−^, calcd 1257.5910) were similar to those of cucumariosides C_1_ (**37**) and C_2_ (**41**), respectively. This may indicate that glycosides **34** and **39** have the same 16β-acetoxyholosta-7-ene aglycone with 22*Z*,24-diene system for **34** and 22*E*,24-diene system for **39**. Actually, these compounds were isolated and their preliminary structures were confirmed by 1D NMR (Figures S6–S8).

Glycosides of group IV (**40** and **36**) have a non-sulfated oligosaccharide chain consisted of glucose, quinovose, and two xylose units. The presence of two Y-type fragment peaks at *m*/*z* 973.4761 [M + Na − Xyl]^+^ and 943.4650 [M + Na − Glc]^+^ in MS/MS of **40** indicates the oligosaccharide chain with two terminal non-methylated monosaccharides. Thus, the structure of the oligosaccharide chain of IV group glycosides was similar to those of I or III groups, without terminal methylated monosaccharide. Fragmentation pattern and HR MS data of compounds **36** (*m*/*z* 1081.5224 [M − H]^−^, calcd 1081.5225) and **40** (*m*/*z* 1081.5221 [M − H]^−^, calcd 1081.5225) corresponded to holostane type aglycones with 16β-OAc and 7(8)-double bond in the nucleus and side chain with two double bonds. Comparison of retention times of these glycosides indicates that **36** have 22*Z*,24-diene system and **40** have 22*E*,24-diene system in the side chains.

The group V (**3**, **4**, **10**, **13**, **21**, **25**, and **29**) include glycosides with branched pentasaccharide chain having one sulfate group at the first xylose unit and 3-*O*-methyl-xylose as a terminal monosaccharide unit and belong to the group of cucumariosides H. Structure of carbohydrate moieties were confirmed by tandem MS. Fragmentation of oligosaccharide chain of the glycosides of group V under collision induced dissociation (CID) conditions gave the intense characteristic product ion series of A-, B- and C-type ions (Table S1). Fragment ions C_4_, B_4_ and A_4_ were mainly observed in desulfated form. In addition, CID spectra of sulfated glycosides provided characteristic product ion ^1,5^A_4_, arising from the cleavage of the ring of sulfated xylose unit. Glycosides **10** (*m*/*z* 1325.5472 [M − Na]^−^, calcd 1325.5478), **13** (*m*/*z* 1181.4692 [M − Na]^−^, calcd 1181.4691), **21** (*m*/*z* 1307.5369 [M − Na]^−^, calcd 1307.5372), **25** (*m*/*z* 1307.5370 [M − Na]^−^, calcd 1307.5372), and **29** (*m*/*z* 1309.5524 [M − Na]^−^, calcd 1309.5529) were identified as cucumariosides H_2_, H_3_, H_5_, H, and H_6_, respectively, based on the comparison of their retention times, MS spectra and elemental compositions with those of standard compounds [33,34,35]. Structures of glycosides **3** and **4** were not defined.

Glycosides of group VI (**6**, **14**, **16**, **26**, **30**, **32**, **42**, and **50**) belong to the group of cucumariosides G, having a linear tetrasaccharide chain with one sulfate group at the first xylose unit and 3-*O*-methyl-xylose as a terminal monosaccharide unit. MS/MS data obtained in both negative and positive ion modes and HR mass values allowed to identify glycosides **14** (*m*/*z* 1193.5051 [M − Na]^−^, calcd 1193.5051) as cucumarioside G_4_ [30], **16** (*m*/*z* 1049.4268 [M − Na]^−^, calcd 1049.4269) as cucumarioside G_2_ [32], **26** (*m*/*z* 1175.4946 [M − Na]^−^, calcd 1175.4950) as cucumarioside G_3_ [31], and **32** (*m*/*z* 1177.5101 [M − Na]^−^, calcd 1177.5106) as cucumarioside G_1_ [29]. Aglycone of **30** (*m*/*z* 1175.4946 [M − Na]^−^, calcd 1175.4950) was identified as 16β-acetoxyholosta-7,22*E*,24-triene-3β-ol. Compound **42** (*m*/*z* 1179.5253 [M − Na]^−^, calcd 1179.5263) probably has holostane type aglycone similar to aglycone of **49** with 16β-OAc and 7(8)-double bond in the nucleus and without double bonds in the side chain. Aglycone of **50** (*m*/*z* 1165.5467 [M − Na]^−^, calcd 1165.5470) is similar to the aglycone of **53** and has the structure 16-acetoxy-20-hydroxy-lanosta-7,24-diene. Structure of the aglycone of glycoside **6** was not defined.

Compounds of group VII (**22** and **27**) have branched oligosaccharide chain consisting of five monosaccharide units—3-*O*-methyl-glucose as a terminal monosaccharide, glucose, quinovose and sulfated xylose in main chain and xylose as branching unit at second monosaccharide. In MS/MS spectra of glycoside **22** (*m*/*z* 1337.5472 [M − Na]^−^, calcd 1337.5478) fragment peaks characteristic for a holostane aglycone with 16β-OAc and two double bonds in the side chain were detected. Obtained data indicated that **27** (*m*/*z* 1339.5629 [M − Na]^−^, calcd 1339.5634) probably has 16β-acetoxyholosta-7,24-diene aglycone.

The group VIII (**17**, **31**, **38**, and **52**) includes compounds with linear tetrasaccharide chain containing terminal 3-*O*-methyl-xylose, sulfated glucose, quinovose and sulfated xylose. These glycosides belong to the group of cucumariosides F. It should be noted that in the positive ion mode it was not possible to obtain stable ions [M + Na]^+^ for disulfated glycosides, so negative ion MS/MS spectra of [M − 2Na]^2−^ ions were used for analysis. Fragmentation of oligosaccharide chain of [M − 2Na]^2−^ ion in MS/MS spectra of disulfated glycosides under CID conditions gave the characteristic product ion series of A-, B- and Y-types ions, which were mainly observed in desulfated form (Table S1). All data allowed identifying glycosides **31** (*m*/*z* 627.2223 [M − 2Na]^2−^, calcd 627.2223) as cucumarioside F_2_ and **38** (*m*/*z* 628.2301 [M − 2Na]^2−^, calcd 628.2301) as cucumarioside F_1_ [36]. Glycoside **17** (*m*/*z* 564.1882 [M − 2Na]^2−^, calcd 564.1882) has 23,24,25,26,27-pentanorlanostane aglycone with an 18(16)-lactone. Structure of the aglycone of glycoside **52** was not defined.

Oligosaccharide chain of glycosides group IX (**11**, **15**, **23**, and **28**) belonging to the group of cucumariosides I, has a main chain with terminal 3-*O*-methyl-xylose, sulfated glucose, quinovose and sulfated xylose and xylose as branching monosaccharide unit. Glycosides **11** (*m*/*z* 702.2493 [M − 2Na]^2−^, calcd 702.2487), **15** (*m*/*z* 630.2098 [M − 2Na]^2−^, calcd 630.2093), **23** (*m*/*z* 693.2437 [M − 2Na]^2−^, calcd 693.2434), and **28** (*m*/*z* 694.2510 [M − 2Na]^2−^, calcd 694.2512) were identified as cucumariosides I_3_, I_4_, I_2_, and I_1_, respectively, based on the comparison of their retention times, MS spectra and elemental compositions with those of standard compounds [37,38].

Besides the described groups, two glycosides with trisaccharide chains were present in analyzed sample. In (−)MS/MS spectra of **33** (group X) (*m*/*z* 1029.4364 [M − Na]^−^, calcd 1029.4371) were detected fragment peaks at *m*/*z* 519.1029 [Glc + Qui + XylSO_3_]^−^, 867.3829 [M − Na − Glc]^−^, and 721.3245 [M − Na − Glc − Qui]^−^. In (−)MS/MS spectra of **35** (group XI) (*m*/*z* 999.4257 [M − Na]^−^, calcd 999.4265) were detected fragment peaks at *m*/*z* 489.0899 [Xyl + Qui + XylSO_3_]^−^ and 867.3832 [M − Na − Xyl]^−^. Both glycosides probably have the same holostane type aglycone with 16β-OAc and two double bonds in side chains (probably 22*E*,24-diene).

Thus, all glycosides of *E. fraudatrix* can be divided into eleven groups in accordance with structures of oligosaccharide chains. Five types of oligosaccharide chains were not found in *E. fraudatrix* previously. According to literature data, the majority of triterpene glycosides of *E. fraudatrix* have 3-*O*-MeXyl as terminal monosaccharide unit. We revealed new glycosides with terminal 3-*O*-MeGlc residue (**22**, **27, 34**, and **39**); two of them (**34**, and **39**) were isolated, and their tentative structures are further supported by 1D NMR (Figures S6–S8).

The fact that not all previously isolated glycosides have been found by LC-MS approach in this study of *E. fraudatrix* could be explained by the changes in the quantitative and qualitative composition of different components of the glycosidic fraction in the samples of one species collected in different places and seasons. A representative example of such changes has been reported for the components of the glycosidic fraction of *Psolus fabricii*, where two different glycosides were predominant or minor in the samples collected near Onekotan Island or Ushishir Islands (Kuril Islands) [41,42]. Another example is given by the glycosides of *Massinium* (=*Neothynidium*) *magnum* where the structures of glycosides from various places or various times of collection were strongly different [43]. In addition, some earlier isolated cucumariosides of A-group may be artifacts formed during the isolation process [24].

**Figure 4:**
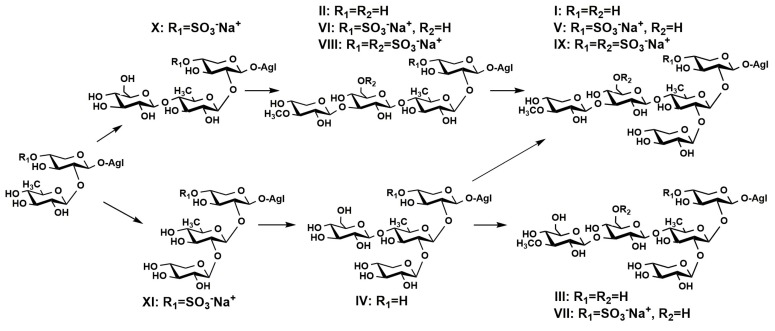
Hypothetic scheme of biosynthesis of oligosaccharide chains in *E. fraudatrix*.

Obtained data allowed us to propose a biosynthetic pathway for oligosaccharide chains in *E. fraudatrix* (Figure 4), in agreement with the biosynthetic pathway of oligosaccharide chains proposed earlier [27]. The elongation of the oligosaccharide chain occurs by the addition of monosaccharide residues to various positions of the forming oligosaccharide chain. This leads to the formation of glycosides with different oligosaccharide chains. Sulfatation of triterpene glycosides may occur at different stages of the forming of carbohydrate chains resulting in the appearance of sulfated oligosaccharide moieties comprised from two to six sugar units. From this viewpoint, cucumarioside B_2_ [27] having the same but non-sulfated carbohydrate chain as **35** represents a biosynthetic precursor of the new glycoside **35**. Analogical relationships are observed in the series of isolated glycosides of *E. fraudatrix*: cucumariosides of C-group (I) → cucumariosides of H-group (V) → cucumariosides of I-group (IX); cucumariosides of A-group (II) → cucumariosides of G-group (VI) → cucumariosides of F-group (VIII); and also in the groups III → VII detected by ESI MS.

### 2.2. Distribution of Detected Glycosides in the Different Body Components

Quantitative and qualitative analysis of detected triterpene glycosides in various body components of *E. fraudatrix* was also performed. We separately extracted triterpene glycoside mixtures from respiratory trees (RT), body walls (BW), gonad tubules (GN), guts (G) and aquapharyngeal bulbs (AB) and analyzed them by LC-ESI QTOF-MS. The profiling revealed that all the triterpene glycosides detected in the whole body extract were also present in all analyzed body parts.

The maximal content of overwhelming majority of the analyzed glycosides was observed in the body walls when compared with other body components of sea cucumber. This observation is a very good corroboration of a defensive role of triterpene glycosides. Since *E. fraudatrix* does not contain Cuvierian tubules, it accumulates the defensive molecules of triterpene glycosides in the body walls in order to indicate to predator its unpalatability. The main components of glycosidic fraction—cucumariosides C_1_ (**37**) and C_2_ (**41**), compounds **39** and **45**, as well as cucumariosides F_1_ (**38**) and G_1_ (**32**)—predominate in the body walls. These compounds contain pentasaccharide non-sulfated or tetrasaccharide mono- or disulfated carbohydrate chains making them highly hydrophilic substances and accelerating their diffusion to the surrounding water. Moreover, such compounds usually demonstrate significant membranolytic activities [1]. All these data also confirm the main external function of glycosides as chemical defense system.

**Figure 5:**
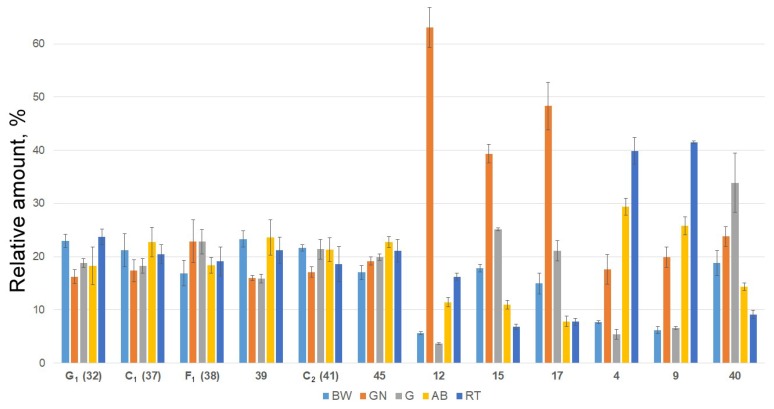
Relative quantities of triterpene glycosides cucumariosides G_1_ (**32**), C_1_ (**37**), F_1_ (**38**) and C_2_ (**41**), and compounds **39**, **45**, **12**, **15**, **17**, **4**, **9**, and **40** in respiratory trees (RT), gonads (GN), aquapharyngeal bulbs (AB), guts (G) and body walls (BW) (bar plots represent the concentration in μg/g animal material of metabolites (mean ± SD) scaled by 100%).

The profiling of extracts from different body components revealed that relative amounts (normalized by sum and scaling) of most compounds were approximately the same (Figure 5; Figure S9). However, several minor compounds were more typical for certain body components. Relative amounts of compounds **12**, **15**, **17** and some others (Figure 5; Figures S9 and S10) are significantly higher in gonads than in body walls or other organs. The analysis of their structures revealed that compounds **15** and **17** have non-holostane aglycones with shortened side chains thus having structural similarity with steroidal hormones of vertebrates. It is known that sex hormones of vertebrates are biosynthesized from cholesterol via the cleavage of its side chain through the oxidation of C-20 and C-22 positions [44]. Actually, compound **12** contains a oxygen-bearing substituent in C-22 position as well as a oxidized C-20 position that makes it a putative biosynthetic precursor of the aglycones with shortened side chains such as **15** and **17**. All these data are in good agreement with the earlier suggested internal biological function of the glycosides—the regulation of oocytes maturation in the sea cucumbers.

Interestingly, the glycosides of group IV (**36** and **40**) having peculiar “undeveloped” branched tetrasaccharide chains without methylated terminal sugar unit were characteristic for guts (Figure S11). There is a correlation between the contents of some glycosides in aquapharyngeal bulbs and respiratory trees (compounds **1**, **2**, **4**, **5**, **7**, **8**, and **9**; Figure S9) that may indicate additional biological functions of triterpene glycosides in the organism-producer, which have to be investigated.

## 3. Materials and Methods

### 3.1. Chemicals

Methanol (quality HPLC gradient grade) and water (quality UV-HPLC grade) were obtained from Panreac (Barcelona, Spain). All other chemicals and reagents were of analytical grade or equivalent. Cucumariosides A_1_–A_4_, A_6_–A_15_ [24,25], H, H_2_–H_8_ [34,35,37], F_1_–F_2_ [36], I_1_–I_4_ [37,38] isolated early from the *E. fraudatrix* and typicoside A_2_ isolated early from the sea cucumber *Actinocucumis typica* [45] were used as standards of triterpene glycosides. Structures of these compounds were established using different methods including high resolution NMR.

### 3.2. Animal Material

Specimens of the sea cucumber *Eupentacta fraudatrix* (Djakonov et Baranova) (family Sclerodactylidae, order Dendrochirotida) were collected at Amursky Bay (Peter the Great Gulf, the Sea of Japan) in April 2017 at a depth of 1.0–1.5 m. Species identification was carried out by Dr. I.Yu. Dolmatov (A.V. Zhirmunsky Institute of Marine Biology, National Scientific Center of Marine Biology, Far Eastern Branch of Russian Academy of Sciences, Vladivostok, Russia). Voucher specimen No. PIBOC-2017-04-EF is preserved in the collection the G.B. Elyakov Pacific Institute of Bioorganic Chemistry.

### 3.3. Sample Preparation and Solid-Phase Extraction (SPE)

Seven fresh animals (m = 3.9 ± 1.3 g) were chopped and extracted thrice with ethanol (totally 0.5 L, for 10 h). Other seven animals were dissected and separated into respiratory trees (RT, m = 1.7 g), body walls (BW, m = 10.2 g), gonad tubules (GN, m = 3.9 g), guts (G, m = 4.3 g) and aquapharyngeal bulbs (AB, m = 3.9 g). Metabolites from five different body components were extracted thrice with ethanol (totally 100 mL, for 10 h). Extracts were filtered, dried and reconstituted in 15 mL ethanol.

Two hundred microliters of the extract was centrifuged, and the supernatant was subjected solid-phase extraction (SPE). SPE cartridges (BondElut C18, 100 mg/1 mL, Agilent Technologies, Santa Clara, CA, USA) were fitted into stopcocks and connected to a vacuum manifold. The sorbent was conditioned with 3 mL of ethanol followed by 3 mL water. Care was taken that the sorbent did not become dry during conditioning. With the stopcocks opened and the vacuum turned on, the samples were loaded onto the cartridge. The 100 μL of extract were loaded into the SPE cartridge by drops. After sample addition, the SPE cartridge was washed with 1 mL of water. Glycosides were eluted with 1 mL of 100% ethanol. These extracts were dried and dissolved in 500 μL 80% MeOH in water (*v*/*v*) and subjected to LC-MS analyses.

### 3.4. LC-MS Analysis

Analysis was performed using an Agilent 1200 series chromatograph (Agilent Technologies, Santa Clara, CA, USA) connected to a Bruker Impact II Q-TOF mass spectrometer (Bruker Daltonics, Bremen, Germany). Zorbax Eclipse XDB-C18 column (1.0 × 150 mm, 3.5 μm, Agilent Technologies, Santa Clara, CA, USA) with Zorbax SB-C8 guard-column (2.1 × 12.5 mm, 5 μm, Agilent Technologies, Santa Clara, CA, USA) were used for chromatographic separation. The mobile phases were 0.1% formic acid in H_2_O (eluent A) and 0.1% formic acid in MeOH (eluent B). The gradient program was as follows: isocratic at 60% of eluent B from start to 3 min, from 60% to 90% eluent B from 3 to 29 min, from 90% to 100% eluent B from 29 to 30 min, isocratic at 100% of eluent B to 35 min, from 100% to 60% eluent B from 35 to 38 min. After returning to the initial conditions, the equilibration was achieved after 15 min. Chromatographic separation was performed at a 0.1 mL/min flow rate at 40 °C. Injection volume was 1 μL.

The mass spectrometry detection has been performed using ESI ionization source. Optimized ionization parameters for ESI were as follows: a capillary voltage of ±4.0 kV, nebulization with nitrogen at 0.8 bar, dry gas flow of 7 L/min at a temperature of 200 °C. Metabolite profiles in positive ion mode were registered using post-column addition of 5 × 10^−4^ M sodium iodide at 60 μL/h flow rate for obtaining stabilized sodium adduct ions. Post-column infusion was performed with syringe pump via T-mixing tee. Based on the results of preliminary experiments, the mass spectra were recorded within *m*/*z* mass range of 100–1500 and 70–1500 for MS/MS spectra (scan time 1 s).

Collision induced dissociation (CID) product ion mass spectra were recorded in auto-MS/MS mode with a collision energy ranging from 75 to 125 eV (an exact collision energy setting depended on the molecular masses of precursor ions). The precursor ions were isolated with an isolation width of 4 Th.

The mass spectrometer was calibrated using the ESI-L Low Concentration Tuning Mix (Agilent Technologies, Santa Clara, CA, USA). Additionally a lock-mass calibration with hexakis (1H, 1H, 3H-tetrafluoropropoxy)phosphazine (922.0098 *m*/*z* in positive mode; 966.0007 *m*/*z* in negative mode; Agilent Technologies, Santa Clara, CA, USA) was performed using Calibrant Reservoir Kit (Bruker Daltonics, Bremen, Germany). The instrument was operated using the otofControl (ver. 4.0, Bruker Daltonics, Bremen, Germany) and data were analyzed using the DataAnalysis Software (ver. 4.3, Bruker Daltonics, Bremen, Germany).

All tests were performed at least in triplicate. The results are expressed as the mean ± standard deviation (SD).

### 3.5. Quantitative Analysis of Detected Triterpene Glycosides in Various Body Component

For quantitative analysis, we used cucumarioside A_1_ from *E. fraudatrix* [24] as a reference standard for non-sulfated glycosides (R_2_ = 0.988), typicoside A_2_ from *Actinocucumis typica* [44] as a reference standard for monosulfated glycosides (R_2_ = 0.999) and cucumarioside I_2_ from *E. fraudatrix* [37] as a reference standard for disulfated glycosides (R_2_ = 0.995). Standards at concentrations of 1.0, 2.5, 5.0, 10.0 and 50 μg/mL were used for building calibration curves (Figures S3–S5). All the experiments were carried out at least three times, LC-MS conditions are identical to those described above. As a result, the amounts of detected compounds were calculated through calibration curves. Results are shown in Table 1 as mean concentration in μg/g animal organs.

For comparative analysis of the content of triterpene glycosides in different organs of *E. fraudatrix* data pretreatment was performed. With the aim to make feature compounds more comparable to each other, the datasets were adjusted using scaling by 100% (for 100%—the total concentration of the compound in all organs). Results are shown in Figure 5 and Figures S9–S11 as the mean ± standard deviation (SD).

## 4. Conclusions

Profiling of *E. fraudatrix* was performed by LC-ESI MS. Analysis of chromatographic behavior, MS and MS/MS data allowed the structural identification of the triterpene glycosides to be performed. Totally, 54 triterpene glycosides were found and structures of 44 constituents were proposed based on LC-ESI MS, chromatographic behavior and biogenetic hypotheses. In accordance with the structures of oligosaccharide chains, all analyzed glycosides of *E. fraudatrix* can be divided into eleven groups, and five of them were found in *E. fraudatrix* for the first time. A theoretical scheme of biogenesis of oligosaccharide chains in the studied species was given. The comparison of the qualitative and quantitative contents from the five different body components revealed that the profiles of some triterpene glycosides differed in the body walls and gonads indicating different external and internal biological functions of these compounds. The predominance of the main highly hydrophilic and membranolytic glycosides of *E. fraudatrix* in the body walls confirms their defensive role. The presence of glycosides in all body components of *E. fraudatrix* indicates their multifunctionality.

## Figures and Tables

**Table 1:** Triterpene glycosides of the ethanol extract of the sea cucumber *E. fraudatrix* detected by LC-ESI MS and their concentrations in different organs.

No. ^a^	R_t_ (min)	Elemental Composition ^b^	Measured *m*/*z*	Molecular Ion Type	Calculated *m*/*z*	Δ (ppm)	Content of Detected Compounds in Different Organs (μg/g) ^c^					Identification (ChemSpider ID)
							BW	GN	G	AB	RT	
**1**	2.0	C_53_H_83_O_28_SNa	1199.4788	[M − Na]^−^	1199.4797	0.8	1.28	2.07	0.74	4.41	5.06	
**2**	2.3	C_58_H_91_O_31_SNa	1315.5265	[M − Na]^−^	1315.5271	0.4	2.06	3.89	2.02	10.76	12.19	
**3**	2.4	C_60_H_93_O_31_SNa	1341.5430	[M − Na]^−^	1341.5427	−0.2	0.58	3.02	0.51	2.15	1.98	
**4**	2.5	C_57_H_85_O_30_SNa	1281.4854	[M − Na]^−^	1281.4852	−0.2	3.12	7.12	2.21	11.88	16.15	
**5**	2.6	C_53_H_83_O_27_SNa	1183.4848	[M − Na]^−^	1183.4848	0.0	8.51	12.85	6.27	33.07	39.82	
**6**	2.7	C_55_H_85_O_27_SNa	1209.4998	[M − Na]^−^	1209.5004	0.5	0.89	4.65	0.61	2.50	2.50	
**7**	2.8	C_52_H_77_O_26_SNa	1149.4426	[M − Na]^−^	1149.4429	0.3	2.30	7.72	2.00	10.60	12.71	
**8**	3.5	C_53_H_82_O_30_S_2_Na_2_	631.2170	[M − 2Na]^2−^	631.2172	0.3	7.23	13.08	5.68	26.16	27.96	
**9**	3.8	C_57_H_86_O_27_	1201.5275	[M − H]^−^	1201.5284	0.7	19.90	63.76	21.23	82.84	132.97	
**10**	4.0	C_60_H_93_O_30_SNa	1325.5472	[M − Na]^−^	1325.5478	0.4	2.05	3.45	2.13	3.96	3.21	Cucumarioside H_2_ * (ID29215132)
**11**	4.3	C_60_H_92_O_33_S_2_Na_2_	702.2493	[M − 2Na]^2−^	702.2487	−0.9	5.40	5.53	5.24	4.21	3.49	Cucumarioside I_3_ * (ID30771157)
**12**	4.3	C_58_H_90_O_26_	1201.5634	[M − H]^−^	1201.5648	1.1	5.98	66.99	3.88	12.14	17.24	
**13**	5.0	C_53_H_81_O_27_SNa	1181.4692	[M − Na]^−^	1181.4691	0.0	67.31	65.70	56.11	38.44	22.47	Cucumarioside H_3_ * (ID29215133)
**14**	5.0	C_55_H_85_O_26_SNa	1193.5051	[M − Na]^−^	1193.5055	0.4	45.93	45.05	51.93	44.31	42.30	Cucumarioside G_4_ ** (ID29216498)
**15**	5.0	C_53_H_80_O_30_S_2_Na_2_	630.2098	[M − 2Na]^2−^	630.2093	−0.7	31.18	68.95	44.00	19.20	11.98	Cucumarioside I_4_ * (ID30771158)
**16**	6.2	C_48_H_73_O_23_SNa	1049.4268	[M − Na]^−^	1049.4269	0.1	182.20	258.47	199.67	104.16	78.53	Cucumarioside G_2_ ** (ID16737749)
**17**	7.7	C_48_H_72_O_26_S_2_Na_2_	564.1882	[M − 2Na]^2−^	564.1882	0.0	164.93	533.31	233.14	86.44	85.75	
**18**	9.1	C_53_H_82_O_24_	1101.5126	[M − H]^−^	1101.5123	−0.2	95.81	79.93	94.56	92.03	77.07	
**19**	9.9	C_62_H_95_O_31_SNa	1367.5580	[M − Na]^−^	1367.5584	0.3	131.88	158.75	151.50	88.37	59.04	
**20**	12.4	C_57_H_87_O_27_SNa	1235.5156	[M − Na]^−^	1235.5161	0.4	87.55	123.77	115.97	76.27	67.76	
**21**	13.0	C_60_H_91_O_29_SNa	1307.5369	[M − Na]^−^	1307.5372	0.2	102.46	101.10	101.29	75.06	56.43	Cucumarioside H_5_ * (ID29212282)
**22**	14.4	C_61_H_93_O_30_SNa	1337.5472	[M − Na]^−^	1337.5478	0.4	39.15	33.91	27.47	30.81	26.60	
**23**	14.5	C_60_H_90_O_32_S_2_Na_2_	693.2437	[M − 2Na]^2−^	693.2434	−0.5	132.94	144.25	135.53	117.55	79.00	Cucumarioside I_2_ *
**24**	15.6	C_57_H_86_O_30_S_2_Na_2_	657.2330	[M − 2Na]^2−^	657.2328	−0.3	164.12	260.64	225.03	129.11	119.00	
**25**	15.7	C_60_H_91_O_29_SNa	1307.5370	[M − Na]^−^	1307.5372	0.2	355.92	300.10	325.16	257.61	205.87	Cucumarioside H **
**26**	16.8	C_55_H_83_O_25_SNa	1175.4946	[M − Na]^−^	1175.4950	0.3	157.60	196.62	189.16	161.61	191.52	Cucumarioside G_3_ ** (ID29213085)
**27**	17.8	C_61_H_95_O_30_SNa	1339.5629	[M − Na]^−^	1339.5634	0.4	24.08	25.54	25.95	22.53	24.23	
**28**	18.1	C_60_H_92_O_32_S_2_Na_2_	694.2510	[M − 2Na]^2−^	694.2512	0.3	188.33	207.58	222.25	202.43	149.95	Cucumarioside I_1_ * (ID30771156)
**29**	19.0	C_60_H_93_O_29_SNa	1309.5524	[M − Na]^−^	1309.5529	0.4	349.25	387.00	349.37	310.11	243.40	Cucumarioside H_6_ * (ID29212283)
**30**	19.1	C_55_H_83_O_25_SNa	1175.4946	[M − Na]^−^	1175.4950	0.3	196.05	245.41	225.43	159.63	183.33	
**31**	20.8	C_55_H_82_O_28_S_2_Na_2_	627.2223	[M − 2Na]^2−^	627.2223	−0.1	650.73	1465.87	1013.44	492.83	518.87	Cucumarioside F_2_ * (ID34981778)
**32**	21.5	C_55_H_85_O_25_SNa	1177.5101	[M − Na]^−^	1177.5106	0.4	1399.21	990.35	1149.58	1114.02	1446.29	Cucumarioside G_1_ ** (ID29212825)
**33**	21.8	C_49_H_73_O_21_SNa	1029.4364	[M − Na]^−^	1029.4371	0.6	3.49	4.46	4.22	3.38	2.41	
**34**	22.4	C_61_H_94_O_27_	1257.5905	[M − H]^−^	1257.5910	0.4	353.39	277.23	270.44	373.37	400.82	
**35**	22.4	C_48_H_71_O_20_SNa	999.4257	[M − Na]^−^	999.4265	0.8	4.00	4.03	4.08	3.80	3.78	
**36**	22.5	C_54_H_82_O_22_	1081.5224	[M − H]^−^	1081.5225	0.1	20.68	29.78	40.22	19.97	12.52	
**37**	23.1	C_60_H_92_O_26_	1227.5799	[M − H]^−^	1227.5804	0.4	2304.76	1883.92	1982.79	2465.98	2218.90	Cucumarioside С_1_ **
**38**	23.2	C_55_H_84_O_28_S_2_Na_2_	628.2301	[M − 2Na]^2−^	628.2301	0.0	1397.27	1896.52	1889.40	1520.29	1581.81	Cucumarioside F_1_ * (ID34981780)
**39**	23.6	C_61_H_94_O_27_	1257.5907	[M − H]^−^	1257.5910	0.2	1232.75	844.94	836.78	1248.59	1121.92	
**40**	23.8	C_54_H_82_O_22_	1081.5221	[M − H]^−^	1081.5225	0.4	78.61	99.33	141.63	60.09	38.25	
**41**	24.2	C_60_H_92_O_26_	1227.5796	[M − H]^−^	1227.5804	0.7	5042.17	3983.86	4978.07	4965.12	4323.97	Cucumarioside С_2_ **
**42**	24.5	C_55_H_87_O_25_SNa	1179.5253	[M − Na]^−^	1179.5263	0.8	107.49	63.87	80.66	86.34	113.97	
**43**	24.6	C_55_H_84_O_22_	1095.5380	[M − H]^−^	1095.5381	0.1	30.86	30.40	35.66	40.90	55.20	Cucumarioside A_5_ ** (ID29214535)
**44**	25.6	C_55_H_84_O_22_	1095.5379	[M − H]^−^	1095.5381	0.2	12.26	15.38	19.73	17.26	25.04	
**45**	25.7	C_60_H_94_O_26_	1229.5956	[M − H]^−^	1229.5961	0.4	3169.77	3565.18	3696.87	4227.02	3920.64	
**46**	26.6	C_47_H_75_O_18_SNa	959.4674	[M − Na]^−^	959.4680	0.6	4.31	5.66	5.78	4.76	6.27	
**47**	27.1	C_55_H_86_O_22_	1097.5534	[M − H]^−^	1097.5538	0.4	128.12	114.38	137.12	144.01	155.36	Cucumarioside A_1_ * (ID29214532)
**48**	27.5	C_55_H_90_O_22_	1101.5851	[M − H]^−^	1101.5851	0.0	38.22	39.78	38.21	45.55	54.26	Cucumarioside A_8_ * (ID29215118)
**49**	27.7	C_60_H_96_O_26_	1231.6110	[M − H]^−^	1231.6117	0.6	328.18	276.91	355.16	406.50	399.65	
**50**	28.5	C_55_H_89_O_24_SNa	1165.5467	[M − Na]^−^	1165.5470	0.3	7.70	6.64	7.32	8.26	9.17	
**51**	28.9	C_55_H_90_O_22_	1101.5843	[M − H]^−^	1101.5851	0.7	19.39	17.88	17.46	18.56	21.60	
**52**	31.3	C_55_H_84_O_27_S_2_Na_2_	620.2328	[M − 2Na]^2−^	620.2326	−0.3	52.92	53.12	56.30	58.02	62.22	
**53**	31.4	C_60_H_98_O_25_	1217.6314	[M − H]^−^	1217.6324	0.9	34.13	32.21	31.91	35.58	44.22	
**54**	32.6	C_56_H_92_O_23_	1131.5951	[M − H]^−^	1131.5957	0.5	19.77	18.64	21.13	23.62	19.24	

^a^ The compound’s numbers correspond to the number of the peaks on (−)LC-MS chromatogram; ^b^ Formula calculated from the accurate mass; ^c^ Concentration is given as μg of compound on g of animal material; BW: body walls; GN: gonads; G: guts; AB: aquapharyngeal bulbs; RT: respiratory trees; * Identification on basis of comparison of retention times, MS/MS data and elemental compositions with corresponding standards; ** Identification based on the elemental compositions and MS/MS data.

## Supplementary Materials

The following are available online at www.mdpi.com/1660-3397/15/10/302/s1, Table S1: Fragmentation of the different types of oligosaccharide chains determined in triterpene glycosides from the ethanol extract of the sea cucumber *E. fraudatrix*, Figure S1: LC-ESI MS base-peak chromatogram (a) and total compounds chromatogram (b) of ethanol extract of sea cucumber *Eupentacta fraudatrix* in negative ion mode, Figure S2: Fragment of ESI MS/MS spectrum of [M + Na]^+^ precursor ion at *m*/*z* 1121 of cucumarioside A_1_, Figure S3: Calibration curve for cucumarioside A_1_ (ion [M − H]^−^ at *m*/*z* 1097), Figure S4: Calibration curve for typicoside A_2_ (ion [M − Na]^−^ at *m*/*z* 1177), Figure S5: Calibration curve for cucumarioside I_2_ (ion [M − 2Na]^2−^ at *m*/*z* 693), Figure S6: ^13^C NMR spectrum of glycoside **39**, Figure S7: ^1^H NMR spectrum of glycoside **39**, Figure S8: ^1^H NMR spectrum of glycoside **34**, Figure S9; Relative quantities of triterpene glycosides detected in *E. fraudatrix* in body walls (BW), gonads (GN), guts (G), aquapharyngeal bulbs (AB), and respiratory trees (RT) (bar plots represent the concentration in μg/g animal material of metabolites (mean ± SD) scaled by 100%), Figure S10: Relative quantities of triterpene glycosides grouped by aglycone structures detected in *E. fraudatrix* in respiratory trees (RT), gonads (GN), aquapharyngeal bulbs (AB), guts (G) and body walls (BW) (bar plots represent the concentration in μg/g animal material of metabolites (mean ± SD) scaled by 100%), Figure S11: Relative quantities of triterpene glycosides grouped by sugar moiety structures detected in *E. fraudatrix* in respiratory trees (RT), gonads (GN), aquapharyngeal bulbs (AB), guts (G) and body walls (BW) (bar plots represent the concentration in μg/g animal material of metabolites (mean ± SD) scaled by 100%).
