# Supplementary material for: Metabolite Profiling of Triterpene Glycosides of the Far Eastern Sea Cucumber Eupentacta fraudatrix and Their Distribution in Various Body Components Using LC-ESI QTOF-MS
Source: Mar Drugs. 2017 Oct 2;15(10):302. doi: 10.3390/md15100302 (PMC5666410; doi:10.3390/md15100302)
Supplement: Supplementary file 1 [file marinedrugs-15-00302-s001.pdf]

Supplementary Materials: **Metabolite profiling of triterpene glycosides of the Far Eastern sea cucumber Eupentacta fraudatrix and their distribution in various body components using LC-ESI QTOF-MS**

Roman S. Popov, Natalia V. Ivanchina, Alexandra S. Silchenko, Sergey A. Avilov, Vladimir I. Kalinin, Igor Yu. Dolmatov, Valentin A. Stonik and Pavel S. Dmitrenok

Contents

Table S1. Fragmentation of the different types of oligosaccharide chains determined in triterpene glycosides from the ethanol extract of the sea cucumber *E. fraudatrix.*

Figure S1. LC-ESI MS base-peak chromatogram of ethanol extract of sea cucumber *Eupentacta fraudatrix* in negative ion mode.

Figure S2. Fragment of ESI MS/MS spectrum of $[M + Na]^+$ precursor ion at *m/z* 1121 of cucumarioside A_1.

Figure S3. Calibration curve for cucumarioside A_1 (ion $[M – H]^-$ at *m/z* 1097).

Figure S4. Calibration curve for typicoside A_2 (ion $[M – Na]^-$ at *m/z* 1177).

Figure S5. Calibration curve for cucumarioside I_2 (ion $[M – 2Na]^{2–}$ at *m/z* 693).

Figure S6. 13C NMR spectrum of glycoside **39**.

Figure S7. 1H NMR spectrum of glycoside **39**.

Figure S8. 1H NMR spectrum of glycoside **34**.

Figure S9. Relative quantities of triterpene glycosides detected in *E. fraudatrix* in body walls (BW), gonads (GN), guts (G), aquapharyngeal bulbs (AB), and respiratory trees (RT) (bar plots represent the concentration in μg/g animal material of metabolites (mean ± SD) scaled by 100%).

Figure S10. Relative quantities of triterpene glycosides grouped by aglycone structures detected in *E. fraudatrix* in respiratory trees (RT), gonads (GN), aquapharyngeal bulbs (AB), guts (G) and body walls (BW) (bar plots represent the concentration in μg/g animal material of metabolites (mean ± SD) scaled by 100%).

Figure S11. Relative quantities of triterpene glycosides grouped by sugar moiety structures detected in *E. fraudatrix* in respiratory trees (RT), gonads (GN), aquapharyngeal bulbs (AB), guts (G) and body walls (BW) (bar plots represent the concentration in μg/g animal material of metabolites (mean ± SD) scaled by 100%).

Table S1. Fragmentation of the different types of oligosaccharide chains determined in triterpene glycosides from the ethanol extract of the sea cucumber *E. fraudatrix*

Type	No	Structure of oligosaccharide chain	$m/z^{-a,\ b}$														
			b_2	b_1	C_4	B_4	$^{1,5}A_4$	$^{1,3}A_4$	C_3	B_3	$^{1,3}A_4/Y_{2\beta}$	$B_3/Y_{2\beta}$	$^{2,5}A_3$	$^{3,5}A_3$	C_2	B_2	B_1
I	**9**, **12**, **18**, **37**, **41**, **45**, **49**, **53**	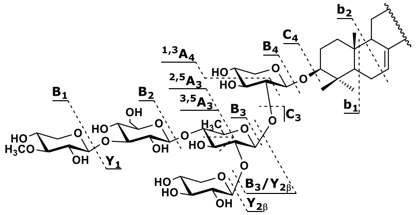	893.36	881.36	759.25	741.24	-	669.22	627.21	609.20	537.18	477.16	417.14	389.14	349.11	331.10	169.05
II	**43**, **44**, **47**, **48**, **51**	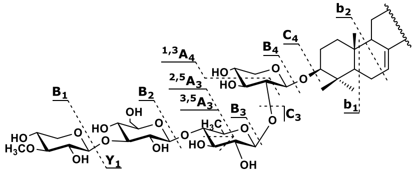	761.32	749.32	627.21	609.20	-	537.18	495.17	477.16	-	-	417.14	389.14	349.11	331.10	169.05
III	**34**, **39**	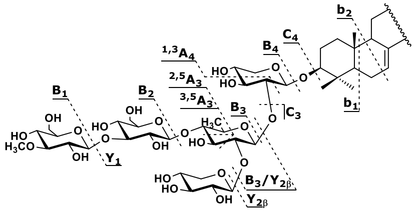	923.37	911.37	789.26	771.25	-	699.23	657.22	639.21	567.19	507.17	447.15	419.15	379.12	361.11	199.06
IV	**36**, **40**	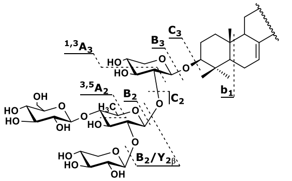	-	735.30	-	-	-	-	613.19	595.18	523.16	-	-	243.08 ($^{3,5}A_2$)	463.14	481.15; 331.10 ($B_2/Y_{2\beta}$)	-
V	**3**, **4**, **10**, **13**, **21**, **29**, **25**	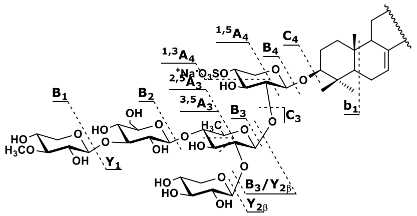	-	983.30	861.19; *741.24*	*723.23*	*695.24*	669.22	627.21	609.20	537.18	477.16	417.14	389.14	-	331.10	169.05

VI	**6**, **14**, **16**, **26**, **30**, **32**, **42**, **50**	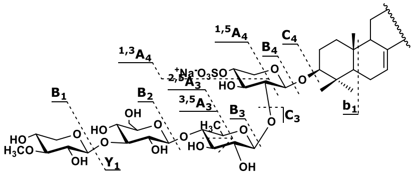	-	*731.31*	729.15; *609.20*	*591.19*	*563.19*	537.18	495.17	477.16	-	-	417.14	389.14	349.11	331.10	169.05
VII	**22**, **27**	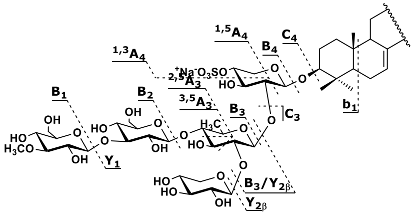	-	*893.36*	891.20; *771.25*	*753.24*	*725.25*	699.23	657.22	639.21	-	507.17	447.15	419.15	-	361.11	-
VIII	**17**, **31**, **38**, **52**	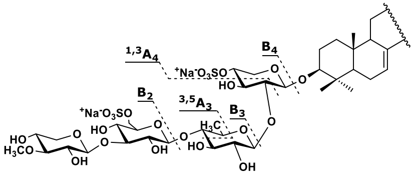	-	-	-	*667.17*	-	593.14	-	533.12	-	-	-	445.10	-	387.06	-
IX	**11**, **15**, **23**, **28**	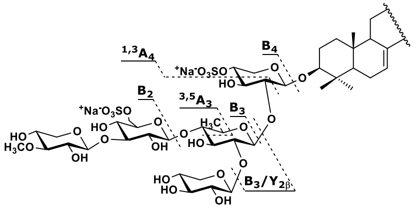	-	-	-	*799.22*	-	725.18	-	665.16	-	533.12	-	445.10	-	387.06	-
X	**33**	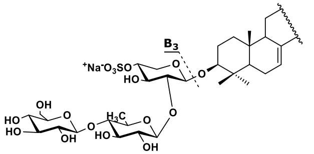	-	-	-	-	-	-	-	519.10	-	-	-	-	-	-	-
XI	**35**	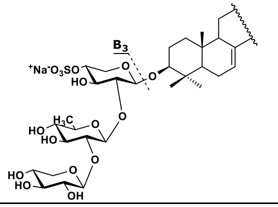	-	-	-	-	-	-	-	489.09	-	-	-	-	-	-	-

[a] Types I-VII: fragment ions from product ion spectrum of $[M + Na]^+$ precursor; types VIII and IX: fragment ions from product ion spectrum of $[M – 2Na]^{2–}$ precursor; types X and XI: fragment ions from product ion spectrum of $[M – Na]^–$ precursor;

[b] Fragment ions C_4, B_4 and A_4 found in desulfated form are in italics.

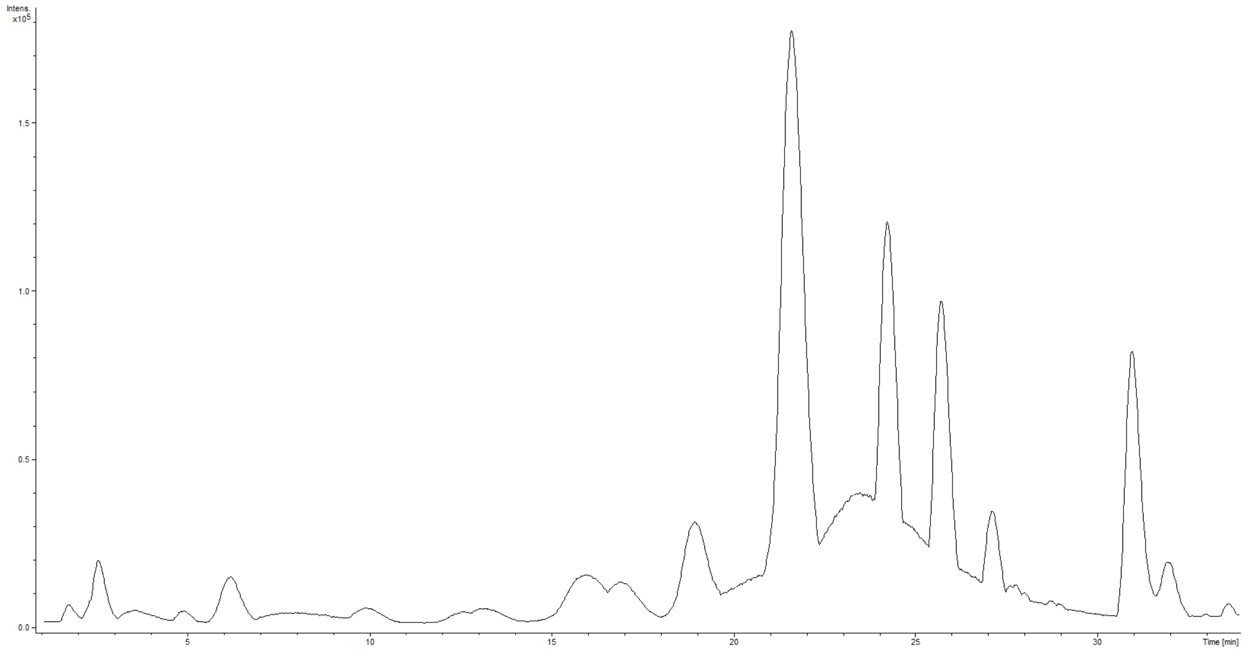

Figure 1S. LC-ESI MS base-peak chromatogram of the ethanol extract of sea cucumber *Eupentacta fraudatrix* in negative ion mode.

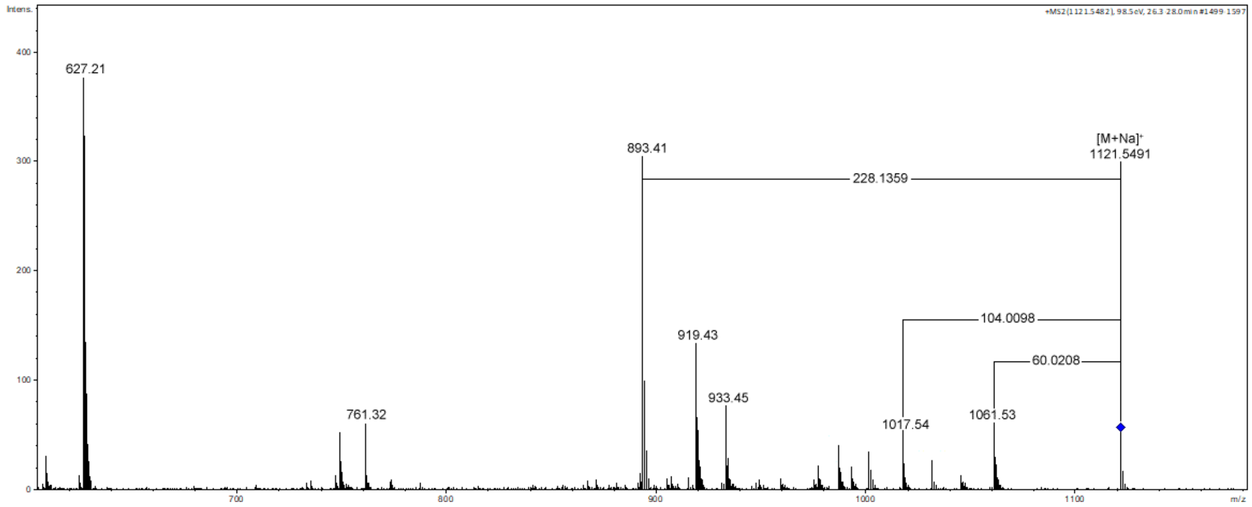

Figure 2S. Fragment of ESI MS/MS spectrum of $[M + Na]^+$ precursor ion at *m*/*z* 1121 of cucumarioside A_1.

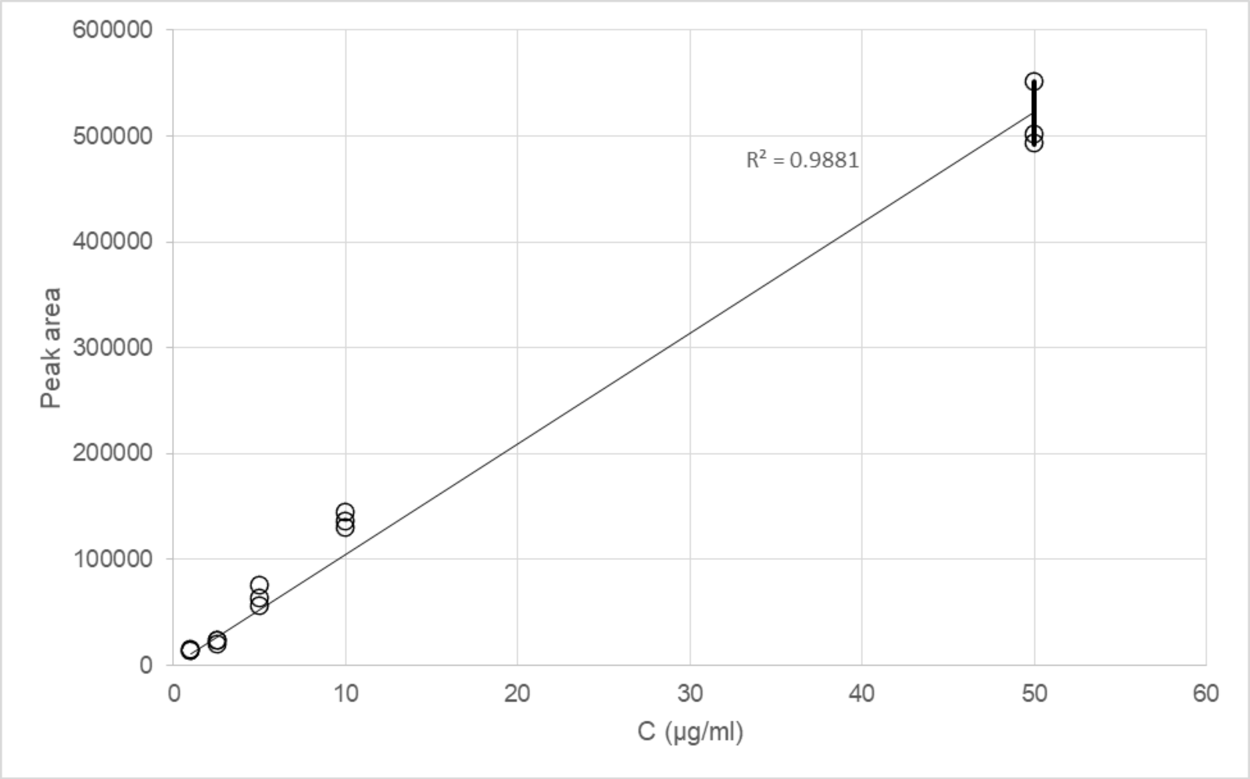

Figure 3S. Calibration curve for cucumarioside A_1 (ion $[M - H]^-$ at *m/z* 1097).

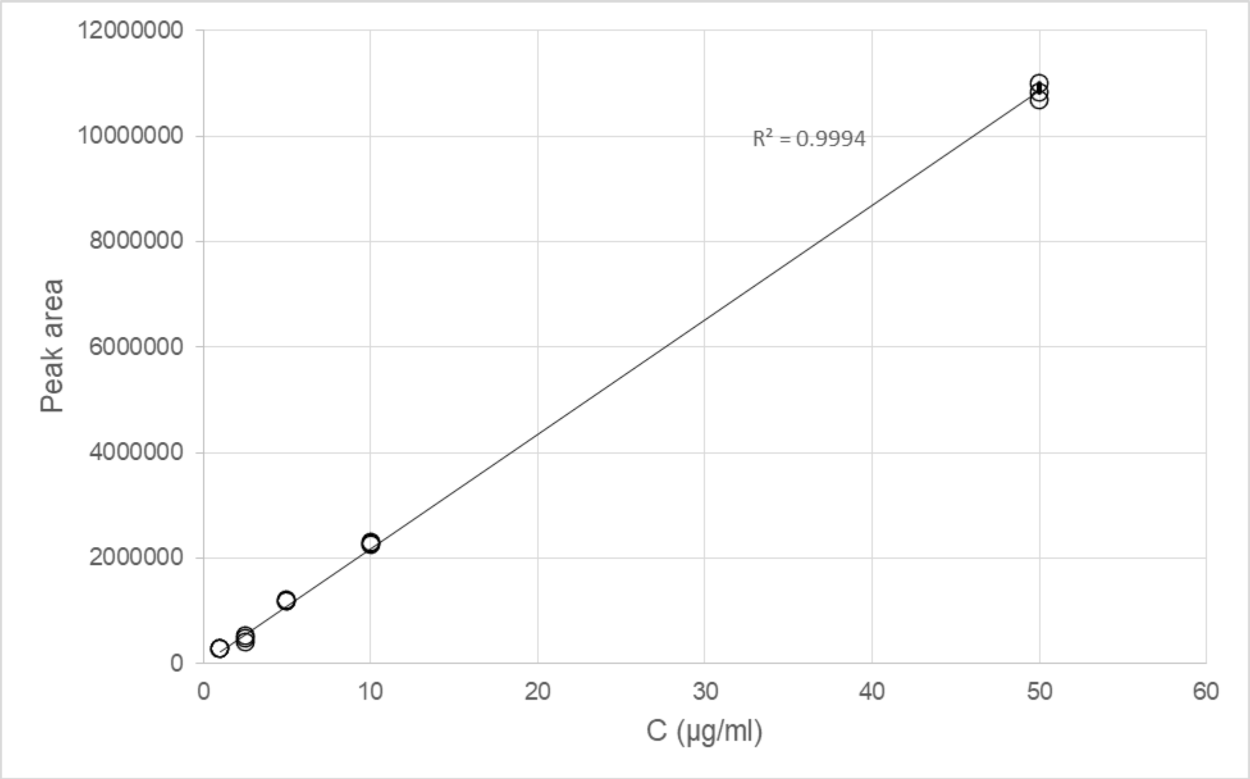

Figure 4S. Calibration curve for typicoside A_2 (ion $[M - Na]^-$ at *m/z* 1177).

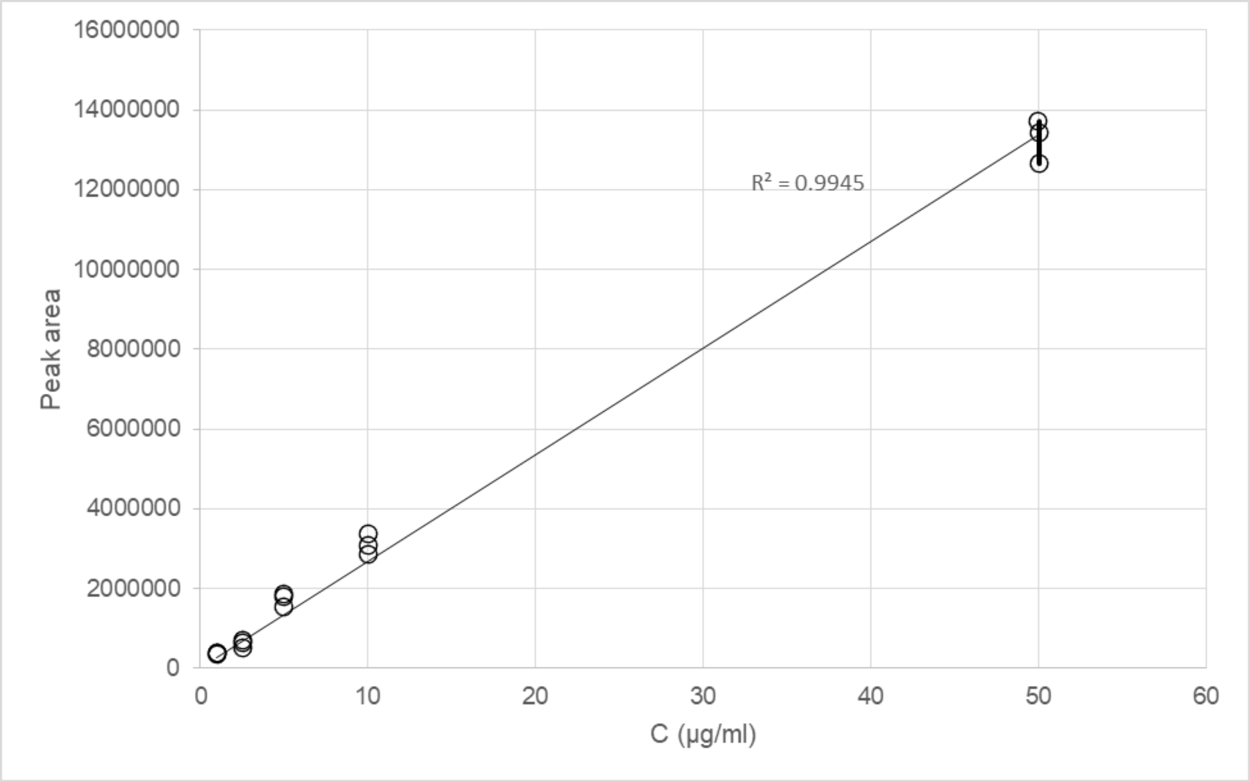

Figure 5S. Calibration curve for cucumarioside I_2 (ion $[M - 2Na]^{2-}$ at *m*/*z* 693).

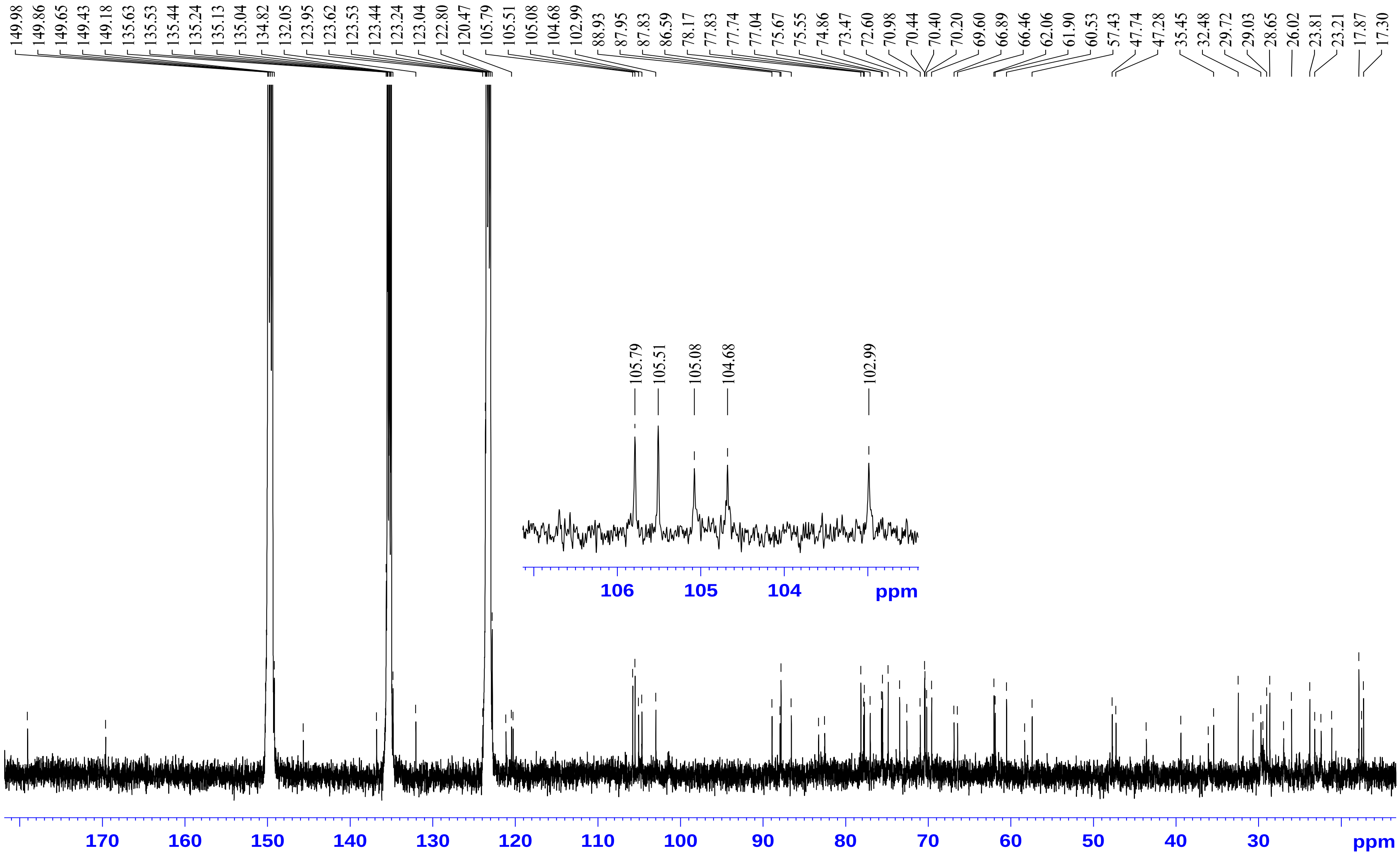

Figure 6S. ^{13}C NMR spectrum of glycoside **39**.

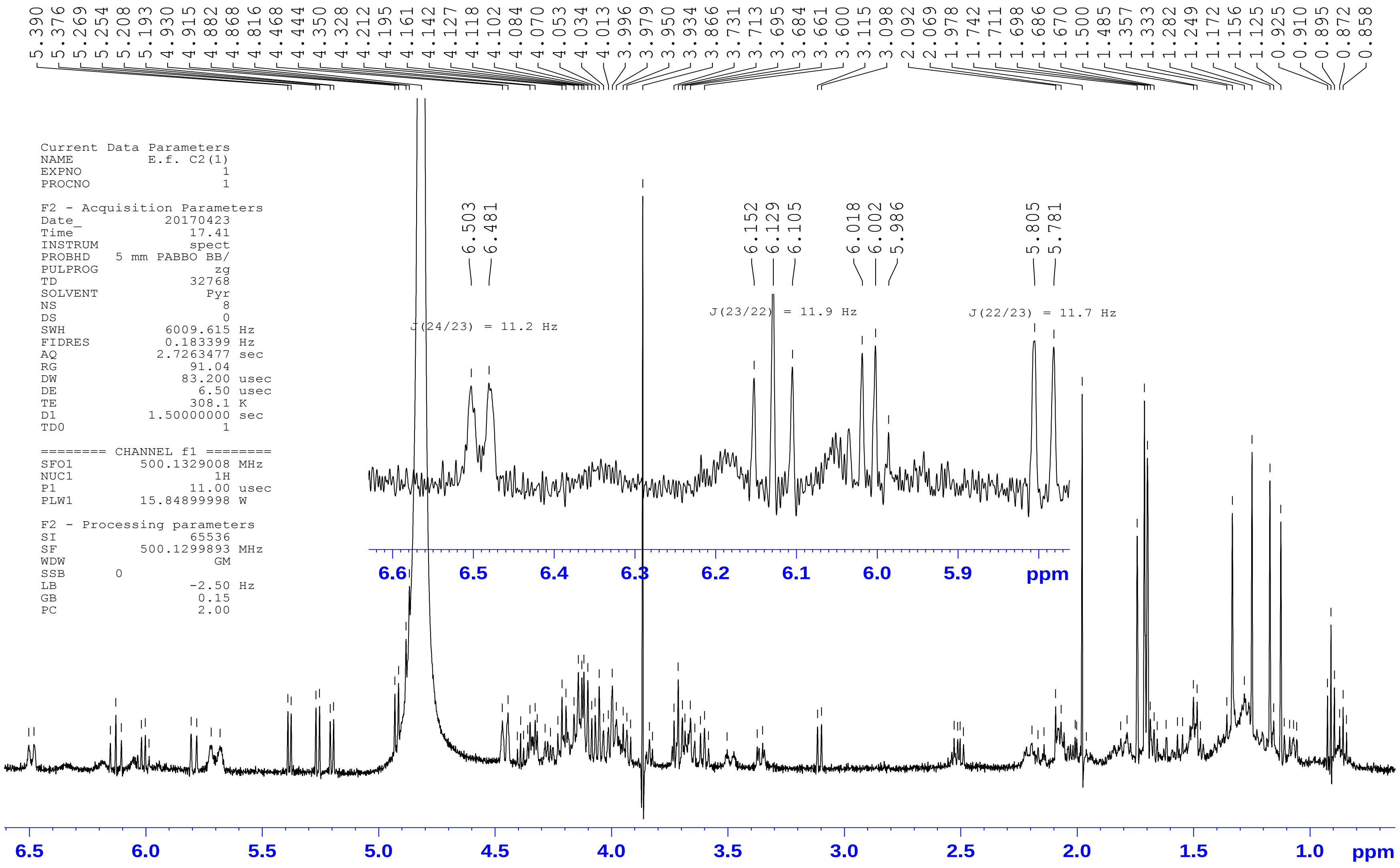

Figure 7S. ^{1}H NMR spectrum of glycoside **39.**

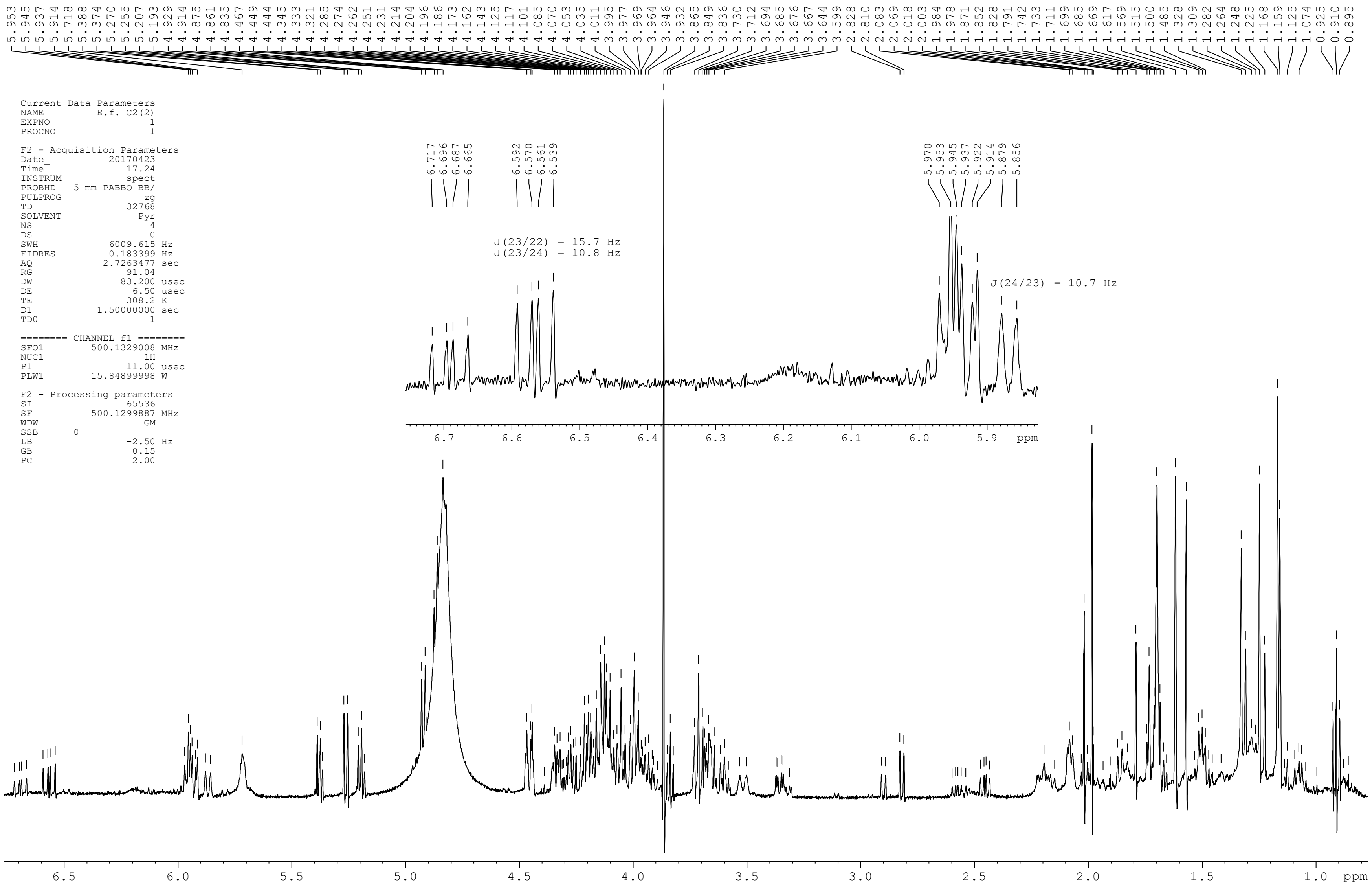

Figure 8S. ^{1}H NMR spectrum of glycoside **34**.

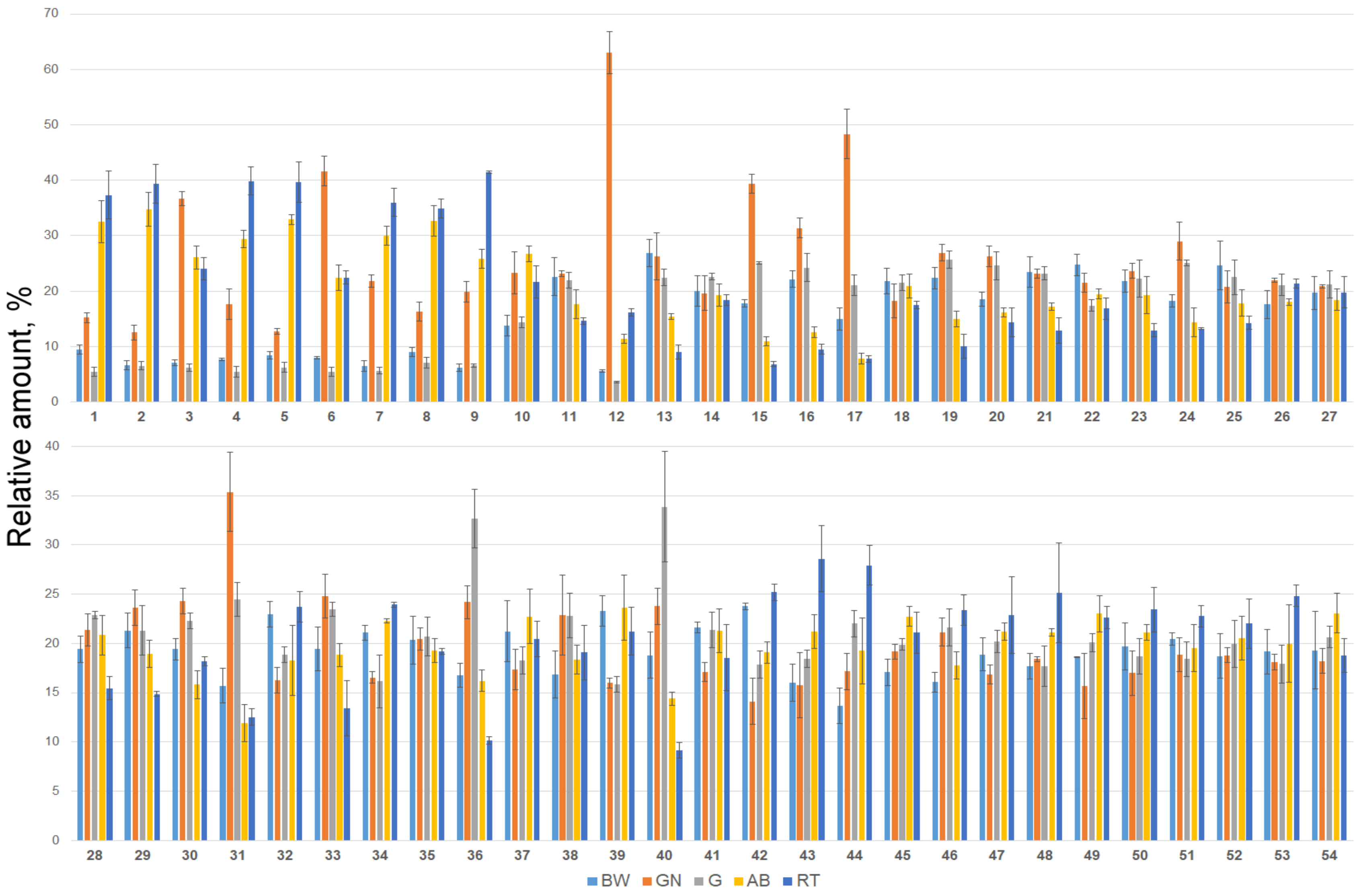

Figure 9S. Relative quantities of triterpene glycosides detected in *E. fraudatrix* in body walls (BW), gonads (GN), guts (G), aquapharyngeal bulbs (AB), and respiratory trees (RT) (bar plots represent the concentration in μg/g animal material of metabolites (mean ± SD) scaled by 100%).

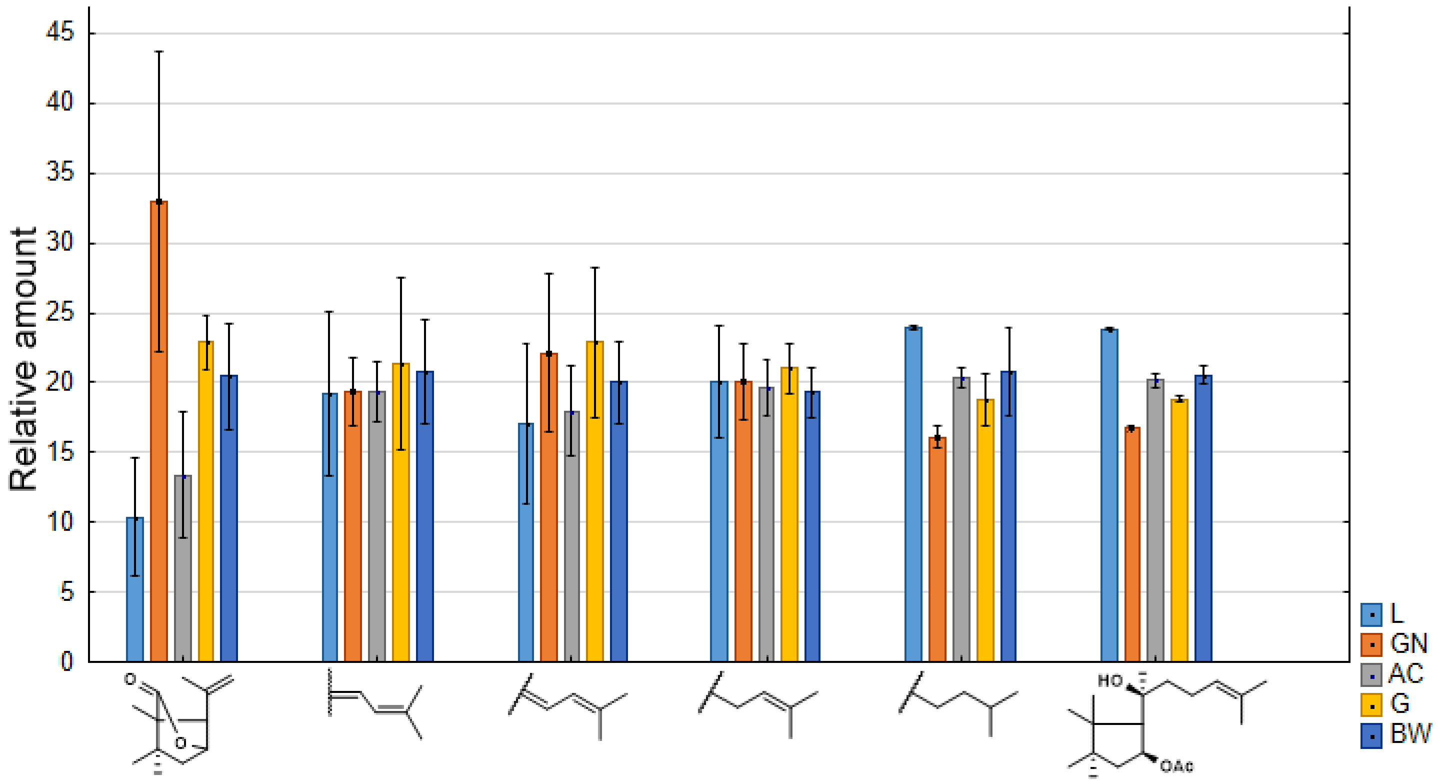

Figure 10S. Relative quantities of triterpene glycosides grouped by aglycone structures detected in *E. fraudatrix* in respiratory trees (RT), gonads (GN), aquapharyngeal bulbs (AB), guts (G) and body walls (BW) (bar plots represent the concentration in μg/g animal material of metabolites scaled by 100%).

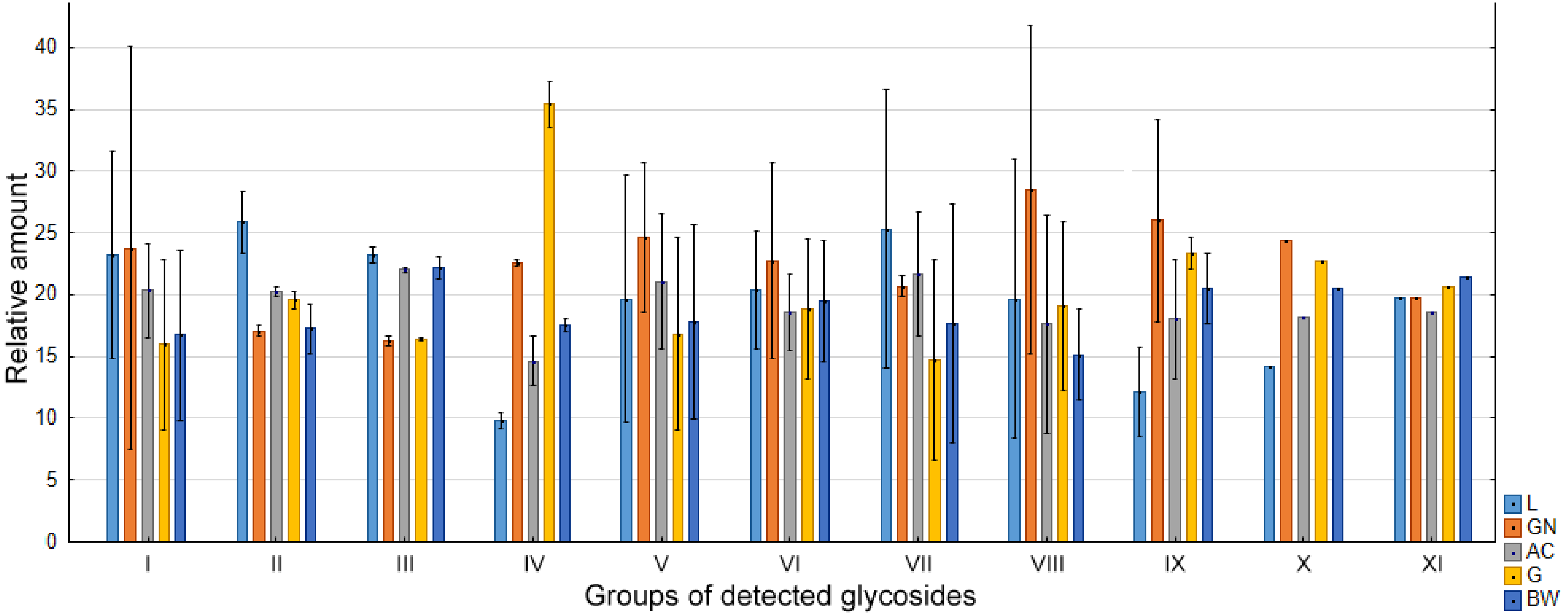

Figure 11S. Relative quantities of triterpene glycosides grouped by sugar moiety structures detected in *E. fraudatrix* in respiratory trees (RT), gonads (GN), aquapharyngeal bulbs (AB), guts (G) and body walls (BW) (bar plots represent the concentration in µg/g animal material of metabolites scaled by 100%).
